# Postoperative serum metabolites of patients on a low carbohydrate ketogenic diet after pancreatectomy for pancreatobiliary cancer: a nontargeted metabolomics pilot study

**DOI:** 10.1038/s41598-019-53287-y

**Published:** 2019-11-14

**Authors:** Chang Moo Kang, BoKyeong Yun, Minju Kim, Mina Song, Yeon-hee Kim, Sung Hwan Lee, Hosun Lee, Song Mi Lee, Seung-Min Lee

**Affiliations:** 1 Division of Hepatobiliary and Pancreatic Surgery, Department of Surgery, Yonsei University College of Medicine, Yonsei Pancreatobiliary Cancer Center, Severance Hospital, Seoul, 03722 Korea; 2 Department of Food and Nutrition, BK21 PLUS Project, College of Human Ecology, Yonsei University, Seoul, 03722 Korea; 3 Department of Systems Biology, University of Texas MD Anderson Cancer Center, Texas, 77030 United States; 4 Department of Nutrition Care, Severance Hospital, Yonsei University Health System, Seoul, 03722 Korea

**Keywords:** Nutrition, Tumour biomarkers

## Abstract

A ketogenic diet is a potential adjuvant cancer therapy that limits glucose availability to tumours while fuelling normal tissues with ketone bodies. We examined the effect of a low carbohydrate ketogenic diet (LCKD) (80% kcal from fat, ketogenic ratio 1.75:1, w/w) compared to a general hospital diet (GD) on serum metabolic profiles in patients (*n* = 18, ≥ 19 years old) who underwent pancreatectomy for pancreatobiliary cancer. Serum samples collected preoperatively (week 0) and after the dietary intervention (week 2) were analysed with a nontargeted metabolomics approach using liquid chromatography–tandem mass spectrometry. Serum β-hydroxybutyrate and total ketone levels significantly increased after 2 weeks of LCKD compared to GD (*p* < 0.05). Principal component analysis score plots and orthogonal partial least squares discriminant analysis also showed significant differences between groups at week 2, with strong validation. In all, 240 metabolites differed between LCKD and GD. Pathways including glycerophospholipid and sphingolipid metabolisms were significantly enriched in the LCKD samples. LCKD decreased C22:1-ceramide levels, which are reported to be high in pancreatic cancer, while increasing lysophosphatidylcholine (18:2), uric acid, citrulline, and inosine levels, which are generally low in pancreatic cancer. Postoperative LCKD might beneficially modulate pancreatic cancer-related metabolites in patients with pancreatobiliary cancer.

## Introduction

Pancreatic cancer causes severe morbidity and has a high mortality rate because its early stage diagnosis is difficult and it presents a high risk of metastasis^1^. The 5-year survival rate (from 2004 to 2010) of patients diagnosed with pancreatic cancer in the United States was only 7%^2^. While only 15–20% of patients with pancreatic cancer are eligible for tumour resection, early detection can increase the 5-year survival rate to ≥ 50%^3^. Surgical treatment for pancreatic cancer commonly leads to malnutrition, which may result in poor surgical outcomes and an increased toxicity of chemo-/radiation therapy; therefore, postoperative nutritional support in patients with pancreatic cancer is important to improve prognosis^4^. Cancer-related malnutrition has been associated with a low quality of life, poor muscle function, increased length of hospital stay, high mortality, and surgical complications^5^. La Torre *et al*. reported that after surgical tumour resection, 88% of patients with pancreatic cancer had a medium-high risk of malnutrition^6^. These patients had significantly longer recovery times and increased morbidity rates when compared to patients who were at a low risk of malnutrition^6^.

A ketogenic diet has recently been investigated as an adjuvant cancer treatment^7^. Allen *et al*. defined the oral caloric composition of a ketogenic diet as 90% fat, 8% protein, and 2% carbohydrate^7^. A ketogenic diet is recognized as an effective dietary intervention for neurological diseases, including epilepsy and Alzheimer’s disease, increasing the levels of ketone bodies such as β-hydroxybutyrate, a major ketone body in blood^8^. The low carbohydrate and high fat composition of a ketogenic diet can limit glucose utilization in cancer cells^7^. Following ketosis, the levels of the gluconeogenic substrates lactate, pyruvate, and alanine decreased in the blood of cachectic cancer patients while free fatty acids and ketone bodies were increased; thus, ketosis may reduce glucose supply to the tumour while nourishing the patient with alternative energy sources^9^. Shukla *et al*. reported that the ketone bodies β-hydroxybutyrate and acetoacetate were associated with inhibited cell growth, reduced glucose uptake, and decreased glutamine and glutamate levels in pancreatic cancer cells; these ketone bodies were also associated with decreased measures of cachexia in patients^10^. In mice with pancreatic cancer, a ketogenic diet (81% kcal from fat) reduced blood glucose levels, increased ketone levels in the blood and tumour, reduced tumour growth, and alleviated cachexia (45% increase in muscle weight, 20% increase in body weight) in comparison to a control diet^10^. Schroeder *et al*. found that 2–5 days of a ketogenic diet in patients with head and neck cancer decreased lactate levels in the tumour, showing that lactate can substitute glucose as an energy source for tumour cells^11^. Fearon *et al*. demonstrated that in patients with gastric cancer, lung cancer, or ovarian cancer, a ketogenic diet (44 kcal/kg/day: 70% of energy from medium-chain triglycerides (MCT)) increased body weight by up to 2 kg after 7 days, with no gastrointestinal side effects, whereas a normal diet (55% kcal from carbohydrates and 31% kcal from fat) did not result in any body weight change after 6 days^9^. Breitkreutz *et al*. reported that among moderately malnourished patients undergoing chemotherapy for colorectal or gastric cancer, an 8-week fat-enriched liquid diet (66% kcal from fat) in addition to normal meals significantly increased body weight and fat-free mass compared to a normal diet^12^. Therefore, a ketogenic diet has a potential therapeutic effect on cancer and may improve the postoperative nutritional status of patients with cancer who are at risk of malnutrition.

Metabolomics is a powerful tool used to identify metabolic changes in the body and to analyse full sets of metabolites in cells, tissues, and organisms^13^. Previous metabolomic analyses have suggested that several blood metabolites are markers for pancreatic cancer. Bathe *et al*. detected increased levels of glutamate, acetone, and β-hydroxybutyrate using ^1^H and 2D nuclear magnetic resonance (NMR) spectroscopy^14^. A metabolomics study using gas chromatography time-of-flight mass spectrometry, liquid chromatography-electrospray ionisation mass spectrometry, and liquid chromatography-tandem mass spectrometry (LC-MS/MS) revealed altered levels of amino acids, fatty acids, bile acids, and lipids such as lysophosphatidylcholine (lysoPC) (18:2), phosphatidylcholine (PC) (34:2), and phosphatidylethanolamine (PE) (26:0) associated with pancreatic cancer^15^. In addition, gas chromatography-mass spectrometry (GC-MS) detected changes in metabolites of patients with pancreatic cancer, such as increases in lactate and asparagine levels and decreases in urea and saturated fatty acid levels^16^, as well as reduced serum 1,5-anhydro-D-glucitol and amino acids, including valine, lysine, and tyrosine^17^. A metabolomics study using flow-injection Fourier transform ion cyclotron resonance mass spectrometry detected changes in serum glycerophospholipids (GPL) in pancreatic cancer patients^18^. Metabolomics can be used to evaluate the effects of a dietary intervention on metabolic profiles of living organisms^19^, and metabolic profiles after dietary intervention have been investigated in patients and in mouse models of cancer^20^. One metabolomics study compared the effects of 6 weeks of a whole grain rye and rye bran-rich diet with a refined white wheat diet in 17 patients with prostate cancer^20^. However, to the best our knowledge, there are no metabolomics studies on the impact of a ketogenic diet in patients who have undergone pancreatectomy for pancreatobiliary cancer.

The purpose of this pilot study was to examine the postoperative effects of a low-carbohydrate ketogenic diet (LCKD) on the serum metabolic profiles in patients who underwent pancreatectomy for pancreatobiliary cancer, compared to a general hospital diet (GD).

## Materials and Methods

### Study subjects

This study was approved by and carried out in accordance with the guidelines and regulations of the Severance Hospital Institutional Review Board (Assignment number: 4-2016-0799) in Seoul, Korea. All subjects provided informed consent. Adult patients (≥ 19 years old) with pancreatobiliary cancer (i.e. pancreatic cancer, duodenal cancer, distal bile duct cancer, or ampullary cancer) who underwent pancreaticoduodenectomy or distal pancreatectomy were enrolled at a pancreatic surgery clinic between November 2016 and May 2017. We excluded patients who were pregnant, illiterate, from a foreign country, or who had severe diabetic complications, hyperlipidaemia with cardiovascular complications, or renal insufficiency with a normal glomerular filtration rate < 90%. After screening 47 patients for eligibility, 30 patients voluntarily enrolled and were randomly assigned to receive GD or LCKD. One patient in the GD group was excluded due to postoperative complications, while 11 patients in the LCKD group were excluded due to postoperative complications (*n* = 4), refusal to consume the diet (*n* = 5), loss of data (*n* = 1), and outlying data (*n* = 1). A total of 18 participants were included in the final analysis (see Supplementary Fig. S1). Body weight and Patient-Generated Subjective Global Assessment (PG-SGA) scores were recorded throughout the study period with the help of a professionally trained dietitian.

### Dietary intervention

Once patients resumed a full liquid oral diet after surgery, they were provided either a GD (n = 9) or an LCKD (n = 9). Both diets were equal in energy content (1500 kcal/d) and were provided for 6 or 7 days during the 2-week hospitalization period before chemoradiotherapy was started. The full liquid and soft GD provided 55–65%, 7–20%, and 15–30% of energy from carbohydrate (C), protein (P), and fat (F), respectively. The full liquid and soft LCKD provided 4%, 16%, and 80% of energy from C, P and F, respectively, targeting a ketogenic ratio of 1.75:1 (F:C + P w/w). Diet initiation differed according to the type of surgery. Patients who underwent a pylorus-preserving pancreaticoduodenectomy were provided a clear liquid diet for 5 days and GD or LCKD from postoperative day (POD) 5. Patients who underwent a distal-pancreatectomy were provided a clear liquid diet until POD 1–3, GD or full liquid LCKD until POD 4, and soft diet until discharge. The protocol was adjusted depending on the patients’ conditions; for example, one GD patient received sips of water from POD 9 for 5 days and resumed oral GD until discharge. Dietary intake was measured through a daily 24-hour recall assisted by a professionally trained dietitian.

### Sample collection

Blood samples were collected under fasting conditions 3 times in total: the day before surgery (week 0), the day of discharge (week 2), and at the first outpatient visit (week 4). Blood samples were centrifuged at 2,500 rpm for 15 min. The supernatant serum was transferred to a clean tube and stored at −80 °C until analysis.

### Quantitative analysis of ketone bodies, insulin, glucose, and TNF-α in blood

The amount of β-hydroxybutyrate (CAS No. 150-83-4, Sigma-Aldrich, USA) in serum was measured by LC-MS/MS using an Ultimate 3000 UHPLC and Q-Extractive Orbitrap Plus (Thermo Fisher Scientific, USA). Standard concentrations ranged from 0.625 to 50 μg/mL. To measure the total ketone (acetoacetate + β-hydroxybutyrate) concentration, the ketone body assay kit (Sigma-Aldrich, USA) was used according to the manufacturer’s instructions. Absorbance was measured at 340 nm using a Tecan GENios multi-functional plate reader (Infinite®F500, TecanGrödig, Austria). Serum insulin levels were measured using an electrochemiluminescence immunoassay with an Insulin Reagent kit (Roche, Germany) and an immunoassay analyser (Roche, Japan). Insulin sensitivity was 0.20 μU/mL. Serum glucose was determined using an enzymatic method (Asan Diagnostics, Korea) according to the manufacturer’s instructions. Serum tumour necrosis factor-α (TNF-α) levels were measured using enzyme-linked immunosorbent assay CyMAX human TNF-α (AbFrontier, Korea) according to the manufacturer’s instructions. The absorbance was measured at 450 nm on a microplate reader (Infinite® 200 PRO, Tecan Trading AG, Switzerland).

### Standard sample preparation for LC-MS/MS analysis

Acetaminophen, sulfadimethoxine, terfenadine, and reserpine (Sigma-Aldrich, Canada) were mixed at the same volume ratio to 10 mg/L in 70% acetonitrile (acetonitrile:methanol = 7:3 v/v) and used as internal standards (IS). Pooled serum samples from all patients were used as quality control (QC) samples. Next, 100 μL of each serum sample was mixed with 800 μL of a solvent (methanol:acetone = 7:3 v/v) and 50 μL of IS. After centrifugation, the clear supernatant was lyophilised in a freeze-dryer for 18 hours at −84 °C, and then received 100 μL of 10% methanol. Finally, 90 μL of the sample was collected and 5 μL of each sample was used in the LC-MS/MS analysis.

### LC-MS/MS analysis

An Ultimate 3000 UHPLC and Q-Extractive Orbitrap Plus was used for the analysis. The column (2.1 × 150 mm) was packed with C18 stationary 1.7 μm-sized resin. The column oven was maintained at 50 °C. Mobile phase A used 0.1% formic acid in water and mobile phase B used 0.1% formic acid in methanol. The total flow rate was 0.4 mL/min and the elution gradient (A/B, v/v) was changed from 100/0 to 0/100 for 15 min, maintained at 0/100 for 4 min, and then changed back to 100/0 for 2 min. The full scan/dd-MS^2^ conditions were as follows: FTMS, ESI-positive mode with a mass resolution of 70,000; full scan range: 80–1000 m/z; dd-MS^2^ (Top 10) resolution of 17,500 with collision energy of 30; flow rate of nitrogen sheath gas and auxiliary gas: 40 (arbitrary units) and 10 (arbitrary units); spray voltage: 3.5 kV; capillary temperature: 320 °C; S- lensRF level: 50; auxiliary gas heater temperature: 300 °C. QC samples were injected into every tenth sample to check data quality and reliability.

### Data processing and metabolite identification

The raw LC-MS data files (.raw) were imported to the XCMS online platform (https://xcmsonline.scripps.edu/) for nonlinear alignment of the data in the time domain and for extraction of the peak intensities^21^. The parameter settings were a 10 sec band width, 15 ppm tolerance for database search, and default values for values not shown. The extracted data included retention time, m/z, and ion intensity. The exact masses of differential ions were verified in online databases, including HMDB (www.hmdb.ca) and MycompoundID (www.mycompoundid.org). The masses and intensities of the query masses were compared with those in the database using a fit score ≥ 0.9. MetaboAnalyst 4.0 (www.metaboanalyst.ca/) was used to conduct hierarchical cluster analysis and draw heat maps to verify the classification ability of the metabolites. Pearson correlation analysis was performed using the same tool. MetaboAnalyst 4.0 is an open access online tool that supports statistical analysis, visualization, and interpretation of metabolomics data^22^.

### Pathway analysis

MetaboAnalyst 4.0 was used to conduct a pathway analysis, taking the concentrations of the identified metabolites into account with multivariate variable importance in the projection (VIP) value > 1.0 and *p* < 0.005. Metabolome view plots were generated to allow identification and analysis of the significantly impacted pathways^23^. Pathways were defined as significantly enriched using cut-offs of *p* < 0.05 for the adjusted false discovery rate (FDR-adjusted) and > 0.1 for the pathway impact score. The KEGG Pathway database (https://www.kegg.jp/kegg/pathway.html) and SMPDB (http://smpdb.ca/) were used to search the superpathway and pathway comprising each individual metabolite. In the case metabolites were involved in multiple metabolic pathways, the most exposed pathway was indicated.

### Statistical analysis

Univariate nonparametric Mann–Whitney *U* tests were run for all metabolites, and multivariate principal component analysis (PCA) and orthogonal partial least squares discriminant analysis (OPLS-DA) were performed for all groups using SIMCA version 14.1 (Umetrics Inc., Sweden)^24–26^. Metabolic peak intensities were log transformed and scaled using Pareto scaling^27^ prior to multivariate analysis using SIMCA 14.1 (Umetrics, Inc., Sweden)^28^. Log transformation was used to normalize right-skewed distribution of metabolite intensity values^29^. Pareto scaling adjusted for the relative importance of large values^27^. Robustness and validity of the results were assessed with parameters R^2^X, R^2^Y, and Q^2^Y, as well as with a cross-validated analysis of variance (CV-ANOVA). The metabolites were filtered as univariate statistical *p*-value < 0.05^30^ and VIP value > 1.0. Information about the patient’s medical and anthropometry measures was expressed as mean ± standard deviation (SD).

## Results

### Patient characteristics, nutritional intake, and blood biochemistry

**Figure 1:**
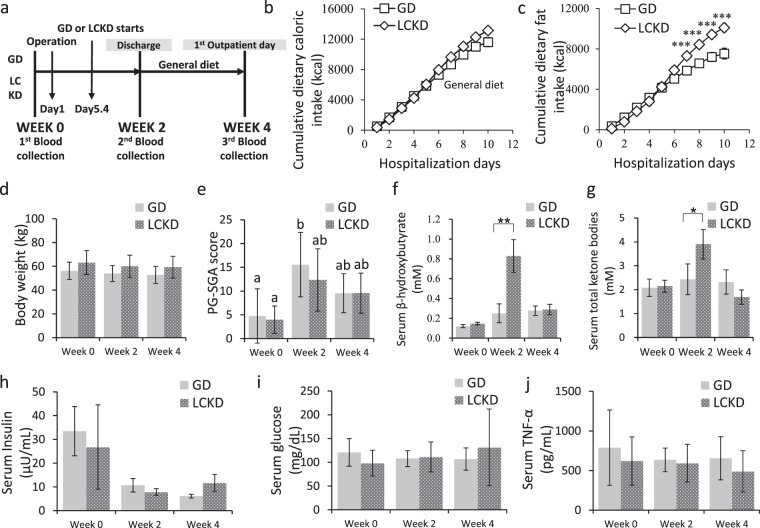
Nutritional changes and blood profiles in GD and LCKD groups during the study period. (**a**) Timeline of the study, (**b**) cumulative total dietary caloric intake, and (**c**) cumulative total dietary fat intake (kcal) are shown. There were no differences in (**d**) body weight (kg) via the Mann-Whitney U test. The PG-SGA score is a nutrition assessment tool that identifies malnutrition in hospitalized patients. Higher scores indicate poorer nutritional statuses. Statistical differences in (**e**) PG-SGA score, serum (**f**) β-hydroxybutyrate, (**g**) total ketone bodies, (**h**) insulin, (**i**) glucose, and (**j**) TNF-α levels were analysed between time periods (general linear model) and between groups (Kruskal Wallis). *p < 0.05; **p < 0.01. Different lowercase letters (a, b) over bars represent significant within group differences (Dunnett T3). Mean ± S.E. PG-SGA, Patient-Generated Subjective Global Assessment; TNF-α, tumour necrosis factor-α.

The general characteristics of participants are summarized in Table 1. Average age, male to female ratio, histological cancer type, and surgical operation type were not significantly different between the groups. The timeline of the study is shown in Fig. 1a. The average postoperative days were not different between GD (17.2 ± 11.8 days) and LCKD (13.4 ± 5.5 days) groups. There was no difference between GD and LCKD groups in the cumulative total caloric intake during the hospitalization period (Fig. 1b). However, the cumulative caloric intake of dietary fat was significantly different between the two groups (Fig. 1c). Body weight was not significantly different between GD and LCKD groups during the study period (Fig. 1d). The PG-SGA score indicated that patients were in a poorer nutrition state after surgery, but this was alleviated at week 4 in both groups, and there were no significant differences between groups (Fig. 1e). At week 2, the LCKD group showed significantly higher ketone levels than the GD group, showing that LCKD induced ketone body production (Fig. 1f,g). At week 4, there were no significant differences in ketone levels compared with the baselines either within each group or between the groups (Fig. 1f,g). Serum insulin, glucose, and TNF-α levels were not different between the GD and LCKD groups during the perioperative period (Fig. 1h–j). Changes in blood chemistry, including blood levels of creatinine, pre-albumin, cholesterol, high-density lipoprotein cholesterol, low-density lipoprotein cholesterol, triglyceride, lipoprotein, transferrin, CEA, and CA19-9, were not significant between the groups during the study period (see Supplementary Table S1).

**Table 1:** General characteristics of GD and LCKD groups.

	GD (*n* = 9)	LCKD (*n* = 9)	*p-*value
Age(year)	66.3 ± 9.8	58.3 ± 7.6	0.072
**Sex(n(%))**			
Male	6(66.7)	5(55.6)	1.000
Female	3(33.3)	4(44.4)	
**Histological type**			
Pancreatic ca.	2	3	
(head/body/tail)	(1/0/1)	(1/2/0)	
Ampulla of Vater cancer	2	1	0.741
Common bile duct	5	3	
Duodenal cancer	0	1	
NET	0	1	
**Surgical operation**			
PPPD	7	8	1.000
DP	2	1	
BMI (kg/m^2^)	22.2 ± 2.7	24.0 ± 2.2	0.149
Weight (kg)	56.3 ± 7.3	63.1 ± 10.1	0.117
CEA	2.13 ± 1.23	1.95 ± 0.63	0.693
CA19-9	150.6 ± 306.5	31.8 ± 33.9	0.265
Hospitalization period	17.4 ± 11.8	11.3 ± 2.1	0.161
Total oral diet period (day)	11.9 ± 10.5	7.5 ± 4.3	0.146
Percentage of energy intake to EER (kcal/kcal %)*			
from total caloric intake (%)	87.5 ± 16.6	89.8 ± 22.4	0.807
from meal (%)	31.9 ± 12.5	44.8 ± 18.4	0.101
from PN	57.0 ± 21.1	45.9 ± 8.5	0.197

Values are mean ± standard deviation. *p*-values were derived from independent Student’s *t*-tests at baseline. The statistical differences between GD and LCKD groups for histological type were obtained using Fisher’s exact test after crossover analysis.

NET, neuroendocrine tumour; PPPD, pylorus-preserving pancreaticoduodenectomy; DP, distal pancreatectomy; BMI, body mass index; CEA, carcinoembryonic antigen; CA19-9, carbohydrate antigen 19-9; EER, estimated energy requirement; PN, parenteral nutrition.

### Non-targeted metabolomics analysis by LC-MS/MS

**Figure 2:**
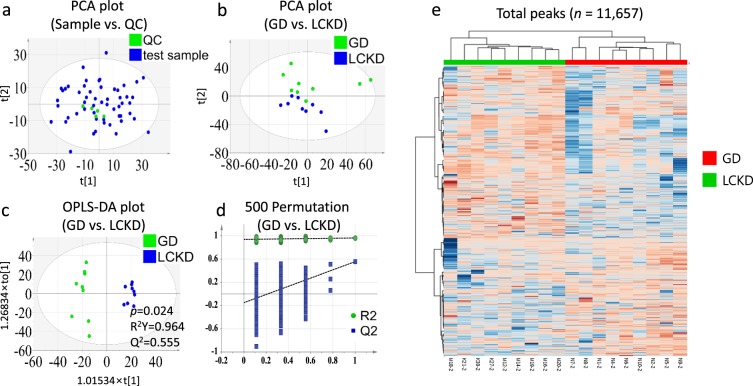
PCA score plot, OPLS-DA three-dimensional score plots and validation plot for the metabolic profiling results between GD and LCKD (week 2). PCA score plots compared (**a**) sample and QC and (**b**) GD and LCKD groups at week 2. (**c**) OPLS-DA score plot (three latent variables, *p* = 0.024, R^2^Y = 0.964, Q^2^ = 0.555) and (**d**) the 500-permutation plot validated GD versus LCKD in the ESI^+^ mode. All permuted R2 and Q2 values on the left were lower than the point on the right and the Q2 regression line had a negative intercept. (R2 = 0.933959, Q2 = −0.174472). (**e**) The total peak intensities between GD and LCKD at week 2 are visualized with a hierarchical cluster analysis heat map. OPLS-DA, orthogonal partial least squares discriminant analysis; PCA, principal component analysis; QC, quality control.

A total of 11,657 ionized compounds in the ESI^+^ mode were detected using LC-MS/MS. While QC samples were closely clustered (Fig. 2a), results for the GD and LCKD were clearly separated at week 2 in two-dimensional PCA score plot analysis (Fig. 2b). OPLS-DA score plot analysis at each time point (weeks 0, 2, and 4) indicated that baseline profiles between the groups were not different (Table 2). A good classification model was detected only between GD and LCKD groups at week 2 (Table 2 and Fig. 2c). A statistical validation of the OPLS-DA model was performed using 500 permutation tests (Fig. 2d). A heat map of the total peaks retrieved from XCMS is presented in Fig. 2e. These results suggested that a differential metabolite profile between GD and LCKD groups was detected only at week 2.

**Table 2:** OPLS-DA parameters and permutation test for distinguishing GD and LCKD groups.

Group	OPLS-DA				*p*-value^b^
	Components^a^	Q^2^	R^2^	Q^2^/R^2^	
**GD-LCKD**					
Week 0–0	5	0.470	0.943	0.50	0.055
Week 2–2	1	0.555	0.964	0.58	0.024
Week 4–4	3	0.608	0.938	0.65	0.057
**GD-GD**					
Week 0–2	6	0.554	0.998	0.60	0.129
Week 0–4	5	0.470	1.000	0.47	0.181
Week 2–4	3	0.569	0.999	0.57	0.174
**LCKD-LCKD**					
Week 0–2	6	0.780	1.000	0.78	0.120
Week 0–4	—	—	—	—	—
Week 2–4	7	0.649	1.000	0.65	0.525

^a^The number of components based on Q^2^ indicates the best classifier of OPLS-DA using a 7-fold cross-validation method.

^b^*p*-value was determined by CV-ANOVA of OPLS-DA in SIMCA 14.1. *p* < 0.05 was considered significant.

CV-ANOVA, cross-validated analysis of variance; OPLS-DA, orthogonal partial least squares discriminant analysis; Q^2^, predictive capability; R^2^, goodness of fit. OPLS-DA not performed was shown as “—”.

### Differential metabolites between GD and LCKD

**Figure 3:**
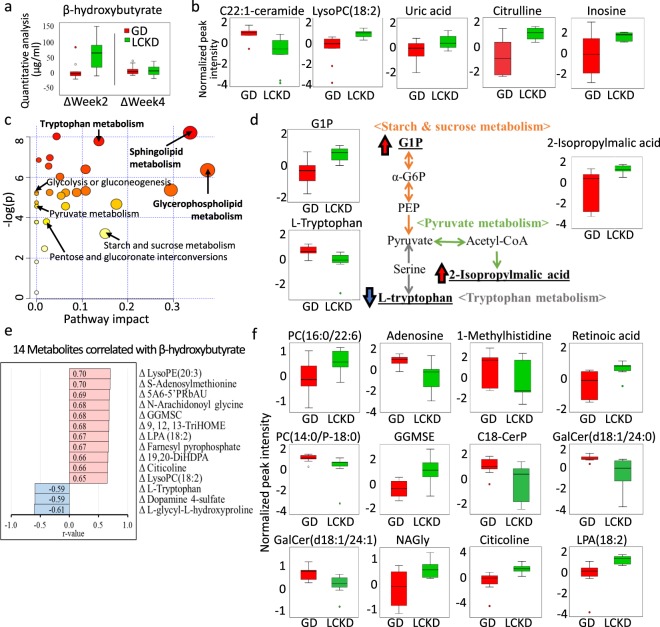
Metabolomic analysis of 240 metabolites enriched in LCKD compared to GD. All figures except Fig. 3a represent data at week 2 compared with baseline. (**a**) Changes in serum β-hydroxybutyrate levels using LC-MS/MS quantitative analysis between baseline and week 2 or 4 are compared in a box plot form. (**b**) Box plots of significantly altered pancreatic cancer-specific biomarkers. Peaks were normalized using log transformation and Pareto scaling. (**c**) An overview of pathway analysis using 65 metabolites (VIP > 1.0, *p* < 0.005) is shown. A total of 33 metabolic pathways were detected by MetaboAnalyst. Significant metabolic pathways (FDR-adjusted < 0.05, pathway impact > 0.1) are labelled in bold and carbohydrate-related pathways are labelled in plain text. (**d**) An overview of carbohydrate-related metabolic pathways among 240 detected metabolites (*p* < 0.05). Among the 65 metabolites (VIP > 1.0, *p* < 0.005) hit in the 3 pathways, those upregulated and downregulated by LCKD are marked with red and blue arrows, respectively. (**e**) Correlation analysis between the 240 metabolites and β-hydroxybutyrate changes (Pattern Hunter, Pearson). Red and blue colours indicate a positive and negative correlation, respectively. (**f**) Box plots of the 4 significant metabolites found in both pathway and correlation analyses. LysoPC, lysophosphatidylcholine; PC, phosphatidylcholine; LPA, lysophosphatidic acid; α-G6P, α-D-Glucose-6P; G1P, Glucose-1-phosphate; PEP, 2-phosphoenolpyruvate; 5A6-5′PRbAU, S-Amino-6-(5′phosphoribitylamino)uracil; GGMSC, Gamma-glutamyl-Se-methylselenocysteine; CerP, ceramide phosphate; GalCer, Galabiosylceramide; NAGly, N-Arachidonoyl glycine.

A slight upregulation in β-hydroxybutyrate was detected by targeted quantitative analysis with LCKD at week 2 compared to GD (*p* = 0.05) (Fig. 3a). To compare the metabolomics data of GD and LCKD at week 2, cut-off values of OPLS-DA VIP score > 1.0 and *p* < 0.05 were used, resulting in the detection of 588 differential peaks. C22:1-ceramide, a previously reported pancreatic cancer-specific metabolite^31^, was significantly downregulated while lysoPC(18:2)^15,18^, uric acid^16^, citrulline^32^, and inosine^15^ were significantly upregulated in the LCKD group at week 2 (Fig. 3b). After excluding drugs (n = 20), xenobiotics (n = 82), a fit score < 0.9 (n = 99), and significantly different metabolites at baseline (n = 147), a total of 240 metabolites were included in further correlation analyses (see Supplementary Table S2).

### Pathway analysis

For metabolic pathway analysis, we chose a more stringent cut-off value of VIP score > 1.0 and *p* < 0.005, resulting in 65 differential metabolites for analysis (Fig. 3c, also see Supplementary Table S3). A total of 8 pathways were found to be significantly enriched, including sphingolipid (FDR-adjusted = 0.003, pathway impact = 0.337) and GPL (FDR-adjusted = 0.005, pathway impact = 0.375) metabolic pathways (Fig. 3c, also see Supplementary Table S3). In addition, carbohydrate-related metabolic pathways were also found among 8 differential metabolites (VIP > 1.0, *p* < 0.05), including glucose-1-phosphate (G1P) and 2-isopropylmalic acid (Fig. 3d).

### Correlation analysis

We conducted a correlation analysis for metabolite changes between weeks 0 and 2 with LCKD-induced changes in β-hydroxybutyrate levels (see Supplementary Table S4). Significant positive and negative correlations were detected in in 63 and 7 metabolites, respectively (see Supplementary Table S4). The top 11 upregulated and 3 downregulated metabolites are presented in Fig. 3e. To further investigate the significance of our LCKD in postoperative patients with pancreatic cancer, metabolites found in both pathway and correlation analyses were selected. Among these metabolites, those with literature-based importance were summarized as box plots in Fig. 3f.

## Discussion

The data demonstrated that our LCKD was ketogenic and induced metabolic alterations in postoperative patients with pancreatobiliary (periampullary and distal pancreatic) cancer who underwent pancreatectomy. A ketogenic diet is generally high in fat, at a ketogenic ratio of 3:1 to 4:1, F:C + P (w/w)^7^. A low glycaemic index treatment and a modified Atkins diet with a 1:1 ketogenic ratio are also used in the Johns Hopkins protocol, which induces ketosis by restricting carbohydrates to 10–20 g/day while not restricting protein, fluid, or energy^33^. Our LCKD targeted a ketogenic ratio of 1.75:1 and a nutritional composition of 15.95 g (4% kcal), 60.00 g (16% kcal), and 132.91 g (80% kcal) of C, P, and F, respectively. To prevent body protein loss, a minimum of 20~25 kcal/kg and 1.0 g of protein per kg was provided, based on the European Society for Clinical Nutrition and Metabolism (ESPEN) guidelines^34^.

LCKD noticeably increased the serum levels of total ketones and β-hydroxybutyrate, suggesting that the ketogenic process was enhanced. In our metabolomics analysis, β-hydroxybutyrate was detected and significantly upregulated by LCKD, but the change was offset at week 4. This supports the ketogenic effect of LCKD. Clinical cancer studies using ketone-producing diets have shown that ketosis may decrease lactate levels in tumour tissues, reduce tumour-associated markers, and improve body weight in cachectic cancer patients and mice^9,10^. The anticancer effects of ketone bodies have received attention due to their ineffectiveness as an energy source for cancer cells with altered mitochondrial function^35^. β-hydroxybutyrate supplementation reduced proliferation in metastatic cancer cells and decreased progression and also increased survival in mice^35^. Recently, a β-hydroxybutyrate-induced reduction of the mTOR oncogenic signalling pathway including c-Myc and its target genes has been reported in pancreatic cancer cells^36^. A β-hydroxybutyrate treatment of pancreatic cancer cells also reduced motility by affecting epithelial-mesenchymal transition markers, and inhibited inflammatory cytokines^36^. In addition, β-hydroxybutyrate has been reported to suppress oxidative stress by increasing oxidative stress resistance factors (FOXO3A and MT2) as a part of the inhibitory actions of class I histone deacetylases^37^. Our LCKD study was the first trial to investigate the effects of a postoperative ketogenic diet in patients with pancreatobiliary cancer who underwent pancreatectomy. Although the 7-day application of LCKD did not prevent body weight loss when compared to the GD, there were significant alterations in serum metabolites, including those associated with pancreatic cancer. This lack of effect of LCKD on body weight might have been due to an insufficient duration of the diet. Previous studies indicated that ketogenic diets lasting 7 days or 8 weeks limited body weight loss in cancer patients that experienced weight loss^7,11^. A well-designed prospective study on the long-term use of a postoperative ketogenic diet in patients with pancreatic cancer is warranted to evaluate the potential role of a ketogenic diet on nutritional statuses perioperatively and in the long-term.

Significant alterations in serum metabolites by LCKD were observed postoperatively in patients with pancreatobiliary cancer. Among significantly altered metabolites, 48.8%, 27.5%, 10.8%, 8.8%, and 2.9% were involved in the metabolism of lipids, amino acids, nucleotides, cofactors and vitamins, and carbohydrates, respectively. Our pathway analysis suggested that changes might have occurred in the metabolic pathways for sphingolipids, tryptophan, ascorbate and aldarate, GPL, thiamine, cysteine and methionine, retinol, and starch and sucrose.

Sphingolipids participate as bioactive lipids within cancer cell signal transduction networks to regulate tumour growth, proliferation, migration, and metastasis^38^. LCKD appeared to lower serum levels of C22:1-ceramide, C18-ceramide phosphate, galabiosylceramides, and glucosylceramides in comparison to GD. Previously, significant elevations of specific individual ceramides, which are metabolites of the sphingolipid pathway, were reported in the blood of cancer patients^18,31,39,40^. Serum C24:6-ceramide^18^ and serum exosome C22:1-ceramide^31^ levels were upregulated in pancreatic cancer. C18-ceramide phosphate is a type of ceramide 1-phosphate (C1P) classified as a sphingolipid. C1P is a key regulator in human pancreatic cancer cell migration and invasion^41^. Galabiosylceramide (d18:1/18:1) was reported to be upregulated in ovarian cancer^42^. In our study, galabiosylceramide (d18:1/24:0) and galabiosylceramide (d18:1/24:1) levels were found to be lowered by the LCKD. Several glucosylceramides were closely linked to anticancer drug resistance^43,44^. Unlike the GD, LCKD might elicit changes in blood ceramide levels against the pancreatic cancer-associated dysregulation of blood metabolites.

GPLs, including PC, PE, PS, phosphatidylinositol, plasmalogen ethanolamine, and plasmalogen choline play structural roles in the lipid bilayers of cell membranes^45^. The GPL metabolic pathway has been associated with pancreatic cancers^46^. PC(16:0/22:6) was downregulated in the serum of patients with pancreatic cancer^18^ but was elevated by our LCKD. PC(14:0/P-18:0), which was upregulated in breast cancer^47,48^, colon cancer, oesophageal cancer, and stomach cancer tissues^48^, was lowered by LCKD. Longnecker *et al*. showed that dietary choline supplementation in rats alleviated pancreatic cancer lesions compared with a choline-free diet^49^. In our study, LCKD increased serum levels of lysoPC(18:2). Reports on lysoPC(18:2) levels in patients with pancreatic cancer are inconsistent, showing reduced serum lysoPC(18:2)^13,18^ and increased plasma lysoPC(18:2)^15^. Moreover, when GPL is degraded, various bioactive lipid mediators are produced, such as phosphatidic acid and lysophosphatidic acid (LPA)^50^. Plasma LPA was elevated in patients with ovarian cancer^51^, but LPA 18:2 was significantly downregulated in human colon cancer tissues compared with non-cancerous tissues^52^. Our LCKD increased LPA 18:2 in patients with pancreatic cancer. Partial reversion of the behaviour of GPL metabolites such as PC(16:0/22:6) and PC(14:0/P-18:0) by LCKD might suggest that LCKD induced some changes in GPL metabolism against pancreatobiliary cancer.

A low carbohydrate and high fat diet is known to reduce glucose and ATP supplies for pancreatic cancer cells, inhibiting their growth and proliferation^10^. Alternatively, ketogenesis may occur with liver glycogen depletion and fatty acid oxidation^53^. We searched for LCKD-induced changes in metabolites of the glucose metabolism. An upregulation of G1P was detected in the LCKD group, which might suggest glycogen degradation by the ketogenic diet-induced elevation in ketone bodies. G1P, derived from glycogen through glycogen phosphorylase, plays an important role in glycolysis, pentose synthesis, ATP generation, and fatty acid synthesis^54^. Another metabolite, 2-isopropylmalic acid, an intermediate of pyruvate metabolism synthesized from acetyl-CoA, was increased by LCKD, although it is downregulated in colon cancer-initiating cells^55^.

Vitamins and amino acid metabolites were also regulated by LCKD. Adenosine, inosine, L-tryptophan, 1-methylhistidine, and creatinine levels were lowered with LCKD. Serum adenosine was upregulated in pancreatic cancer patients^18^. Inosine, detected by GC- and LC-MS-based metabolomic analyses, was upregulated in plasma samples from patients with pancreatic ductal adenocarcinoma^15^. Serum tryptophan was upregulated in a pancreatic cancer mouse xenograft and 1-methylhistidine was upregulated in murine pancreatic tumour tissue^46^. In patients with pancreatic cancer, the serum creatinine level was found to be elevated using ^1^H NMR spectroscopy metabolomic profiling^56^ but was found to be decreased when analysed by GC-MS^17^. However, retinoic acid, N-arachidonoyl glycine, gamma-glutamyl-Se-methylselenocysteine, citicoline, uric acid, and citrulline levels were elevated with LCKD. The increased level of retinoic acid was associated with cell cycle arrest and a synergistic effect on apoptosis when combined with chemotherapeutic treatment in pancreatic cancer cells^57^. N-Arachidonoyl glycine exhibited anti-inflammatory effects in rat ear oedema and peritonitis models^58^. Gamma-glutamyl-Se-methylselenocysteine, an organic selenium compound, showed an anti-cancer effect in a rat model of colon cancer^59^. A protective effect of citicoline on colitis has been reported in rats, related to its contribution to anti-inflammatory and antioxidant mechanisms^60^. Uric acid was downregulated in patients with pancreatic cancer compared with healthy participants^16^. Citrulline was decreased in patients with pancreatic cancer in an LC-MS-based plasma profiling study^32^. Overall, LCKD might partially provide beneficial effects against pancreatic cancer.

Animal studies have shown increased survival rates with a ketogenic versus non-ketogenic diet; however, few human clinical studies have investigated this^61^. Anti-cancer effects of ketogenic diets were evidenced in animal studies by a clear reduction in tumour mass^61^. A ketogenic diet (C:P:F = 0.76%:8.36%:78.8%) reduced tumour growth and improved survival compared to a standard chow diet (composition unknown) in glioma cancer cell-implanted mice (median survival days: ketogenic diet = 25 days, standard diet = 19 days)^62^. A ketogenic diet supplemented with omega-3 fatty acids and MCTs (C:P:F = 0.2%:13.0%:35.5%) delayed tumour formation in human gastric cancer cell-implanted mice compared with a standard diet (C:P:F = 36.4%:23.8%:7.0%) (g/100 g diet)^63^. LCKD might provide anti-cancer effects by reducing the proliferation of any residual postoperative cancer cells. Based on the present study, a prospective randomized clinical trial evaluating the impact of LCKD on survival in patients with pancreatobiliary cancers is necessary.

Although the present study was a phase I prospective clinical trial demonstrating the safety and feasibility of the postoperative use of an LCKD and its effects on the metabolomics of patients with pancreatobiliary cancer, there are several limitations to this study. First, the study population is small due to the high exclusion and withdrawal rates. The measurement of ketone bodies might have provided more insight if more frequent and using other sources (e.g. urinary). The present metabolomic analysis was highly validated by the OPLS-DA model, suggesting that our results show significant differences between GD and LCKD. As a novel metabolomics study on the application of a post-surgery dietary intervention in patients with cancer, our results may provide useful data for further research.

In summary, our study successfully demonstrated that LCKD with 80% kcal from fat induced moderate ketosis by increasing postoperative serum total ketones and β-hydroxybutyrate in patients with cancer. In addition, an LCKD might revert some pancreatic cancer metabolite biomarkers, such as C22:1-ceramide, lysoPC (18:2), uric acid, citrulline, and inosine, and might also affect the metabolism of carbohydrates, amino acids, and vitamins. The LCKD used in our study might provide potential benefits to patients with pancreatobiliary cancer who undergo pancreatectomy. Further studies are mandatory to ascertain the effects of the serum metabolite changes induced by LCKD.

## Supplementary information

Supplementary Figure S1 and Tables S1, S2, S3, S4

## Data Availability

All data generated or analysed during this study are included in this published article and its Supplementary Information File.
