# Supplementary material for: Postoperative serum metabolites of patients on a low carbohydrate ketogenic diet after pancreatectomy for pancreatobiliary cancer: a nontargeted metabolomics pilot study
Source: Sci Rep. 2019 Nov 14;9:16820. doi: 10.1038/s41598-019-53287-y (PMC6856065; doi:10.1038/s41598-019-53287-y)
Supplement: Supplementary file 1 — Supplementary Figure S1 and Tables S1, S2, S3, S4 [file 41598_2019_53287_MOESM1_ESM.docx]

Postoperative serum metabolites of patients on a low carbohydrate ketogenic diet after pancreatectomy for pancreatobiliary cancer: a nontargeted metabolomics pilot study

Chang Moo Kang^1^, BoKyeong Yun^2^, Minju Kim^2^, Mina Song^2^, Yeon-hee Kim^2^, Sung Hwan Lee^3^, Hosun Lee^4^, Song Mi Lee^4^, Seung-Min Lee^2^*

^1^Division of Hepatobiliary and Pancreatic Surgery, Department of Surgery, Yonsei University College of Medicine, Yonsei Pancreatobiliary Cancer Center, Severance Hospital, Seoul 03722, Korea.

^2^Department of Food and Nutrition, BK21 PLUS Project, College of Human Ecology, Yonsei University, Seoul 03722, South Korea.

^3^Department of Systems Biology, University of Texas MD Anderson Cancer Center, Texas 77030, United States.

^4^Department of Nutrition Care, Severance Hospital, Yonsei University Health System, Seoul 03722, South Korea.

* Correspondence and request for reprints: Seung-Min Lee

Department of Food and Nutrition, College of Human Ecology, Yonsei University,

50 Yonsei-ro, Seodaemun-gu, Seoul 03722, South Korea

Tel: 82-2-2123-3118; Fax: +82-2-2123-3115; E-mail: leeseungmin@yonsei.ac.kr

**Supplementary Figure S1. Flow chart of study population and scheme of the study.**

**
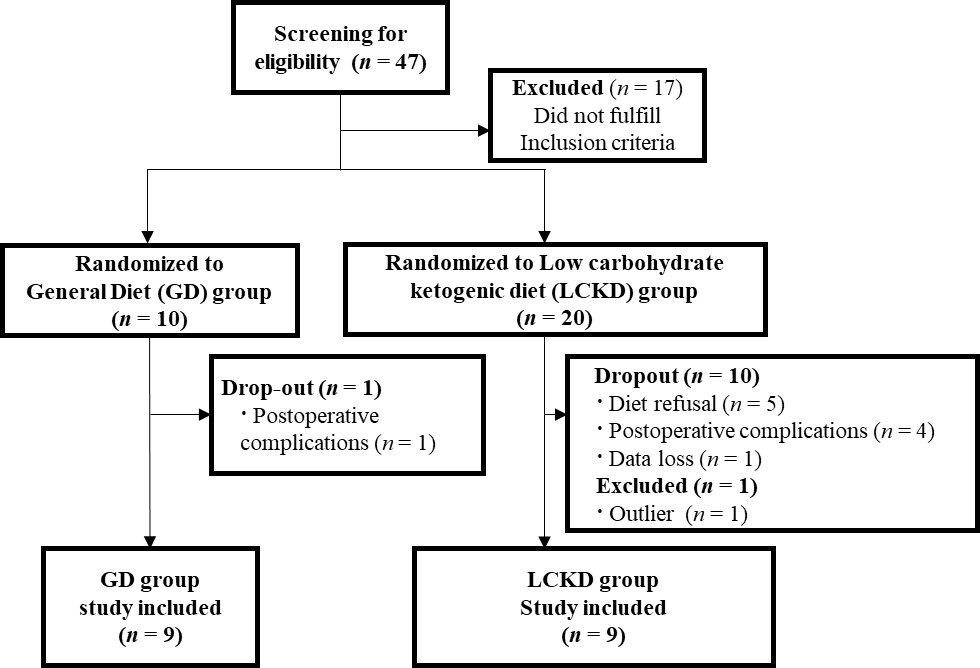
**

**Supplementary Table S1. Comparison of blood biochemistry parameters between GD and LCKD groups.**

|  | Reference range | GD (n=9) | | | | LCKD (n=9) | | | | *p-v*alue^§^ |
| --- | --- | --- | --- | --- | --- | --- | --- | --- | --- | --- |
|  |  | Week 0 | Week 2 | Week 4 | *p-v*alue^#^ | Week 0 | Week 2 | Week 4 | *p-v*alue^#^ |  |
| Creatinine (mg/dL) | 10-300 | 65.4±54.5 | 82.3±45.7 | 150.2±49.3^*^ | 0.004 | 131.8±87.7 | 83.0±38.3 | 232.0±93.5^*^ | 0.003 | 0.563 |
| Pre-albumin (g/L) | 150-350 | 198.9±61.2 | 153.3±70.3 | 226.9±69.0 | 0.064 | 267.9±74 | 151.1±44.7^**^ | 228.4±78.1 | 0.005 | 0.826 |
| Cholesterol (mg/dL) | 200 | 155.8±46.2 | 136.7±30.4 | 156.0±48.5 | 0.561 | 184.7±46.1 | 147.2±20.4^*^ | 180.0±27.1 | 0.045 | 0.433 |
| HDL-C (mg/dL) | ≥ 60 | 43.9±11.3 | 27.8±9.4^**^ | 42.4±6.9 | 0.003 | 44.4±9.7 | 25.4±4.07^**^ | 39.6±8.53 | 0.000 | 0.806 |
| LDL-C (mg/dL) | <100 | 86.0±36.5 | 77.6±29.5 | 97.4±41.9 | 0.551 | 112.9±38.6 | 88.7±18.9^*^ | 112.0±27.6 | 0.163 | 0.607 |
| TG (mg/dL) | <150 | 120.8±74.5 | 130.4±67.3 | 110.6±51.2 | 0.819 | 137.9±89 | 184.9±111 | 131.0±49.7 | 0.390 | 0.292 |
| Lipoprotein (mg/dL) | >30 | 20.0±13.9 | 18.1±14.1 | 26.2±26.5 | 0.658 | 18.8±11.8 | 16.0±9.22 | 27.0±19.2 | 0.245 | 0.906 |
| Transferrin (mg/dL) | 212-360 | 208.7±53.5 | 181.4±53.1^*^ | 221.4±42.7 | 0.160 | 233.7±26.1 | 166.3±36.8^**^ | 227.9±50.6 | 0.001 | 0.562 |
| CEA (ng/mL) | <3.00 | 2.13±1.23 | 6.91±17.1 | - | 0.419^γ^ | 1.95±0.63 | 1.32±0.37 | - | 0.002^γ^ | 0.906^ǂ^ |
| CA19-9 (U/mL) | <37.0 | 150.6±306.5 | 41.3±44.7 | - | 0.259^γ^ | 31.8±33.9 | 15.5±4.81 | - | 0.156^γ^ | 0.103^ǂ^ |

Values are mean ± standard deviation. ^#^Differences in time within a group were tested by the randomized block design of time. ^§^ Differences between the GD and LCKD were tested by the repeated measure ANOVA by time and groups ^γ^Differences between week 0 and week 2 were compared with a paired t-test ǂ Difference between groups and time were compared by linear regression analysis.

^*^ p<0.05, ^**^ p<0.005 means statistically significant difference in baseline (Paired t-test).

HDL-C, high-density lipoprotein cholesterol; LDL-C, low-density lipoprotein cholesterol; TG, triglyceride; ANOVA, analysis of variance; GD, general diet; LCKD, low-carbohydrate ketogenic diet

**Supplementary Table S2. Database information and statistical analysis of 240 metabolites significantly altered at week 2 by LCKD.**

| **No.** | **m/z** | **RT**  **(min)** | **Metabolite** | **HMDB** | **VIP** | ***p*-value** | **Log_10_**  **FC** | **Superpathway** | **Pathway** | **Correlation** | |
| --- | --- | --- | --- | --- | --- | --- | --- | --- | --- | --- | --- |
|  |  |  |  |  |  |  |  |  |  | **r** | **p** |
| 1 | 179.0001 | 21.91 | L-Gulonolactone | HMDB03466 | 1.29 | 0.004 | 0.49 | Carbohydrate | Ascorbate and aldarate metabolism | 0.13 | 0.618 |
| 2 | 193.0081 | 21.85 | Diketogulonic acid | HMDB05971 | 1.16 | 0.002 | 1.27 | Carbohydrate | Ascorbate and aldarate metabolism | 0.47 | 0.051 |
| 3 | 287.1138 | 5.60 | Salicin | HMDB03546 | 1.49 | 0.035 | -1.38 | Carbohydrate | Glycolysis / Gluconeogenesis | 0.11 | 0.669 |
| 4 | 177.0870 | 1.00 | 2-Isopropylmalic acid | HMDB00402 | 2.20 | 0.005 | 0.82 | Carbohydrate | Pyruvate metabolism | 0.25 | 0.315 |
| 5 | 380.2130 | 5.60 | S-Lactoylglutathione | HMDB01066 | 1.63 | 0.043 | 1.41 | Carbohydrate | Pyruvate metabolism | 0.50 | 0.033 |
| 6 | 321.0856 | 4.57 | Octanoylglucuronide | HMDB10347 | 1.91 | 0.005 | 2.10 | Carbohydrate | Starch and sucrose metabolism | 0.62 | 0.006 |
| 7 | 261.1446 | 6.73 | Glucose 1-phosphate | HMDB01586 | 1.33 | 0.004 | 1.10 | Carbohydrate | Starch and sucrose metabolism | 0.52 | 0.027 |
| 8 | 113.9765 | 2.48 | Creatinine | HMDB00562 | 2.02 | 0.008 | -1.46 | Amino acid | Arginine and proline metabolism | -0.20 | 0.428 |
| 9 | 241.0052 | 1.98 | Homocarnosine | HMDB00745 | 1.99 | 0.004 | 1.50 | Amino acid | Arginine and proline metabolism | 0.65 | 0.004 |
| 10 | 116.0530 | 3.80 | L-Proline | HMDB00162 | 1.31 | 0.029 | -0.87 | Amino acid | Arginine and proline metabolism | -0.08 | 0.760 |
| 11 | 104.0709 | 0.59 | Gamma-Aminobutyric acid | HMDB00112 | 1.25 | 0.023 | -0.40 | Amino acid | Arginine and proline metabolism | -0.07 | 0.779 |
| 12 | 176.0518 | 2.49 | Citrulline | HMDB00904 | 3.05 | 0.004 | 2.44 | Amino acid | Arginine metabolism | 0.38 | 0.122 |
| 13 | 203.1390 | 8.48 | Dimethyl-L-arginine | HMDB01539 | 1.89 | 0.004 | 2.36 | Amino acid | Arginine metabolism | 0.19 | 0.452 |
| 14 | 338.0679 | 1.46 | Kyotorphin | HMDB05768 | 1.51 | 0.019 | 0.81 | Amino acid | Arginine metabolism | 0.53 | 0.024 |
| 15 | 356.2278 | 7.77 | S-Adenosylmethioninamine | HMDB00988 | 2.34 | 0.002 | 2.27 | Amino acid | Cysteine and methionine metabolism | 0.53 | 0.023 |
| 16 | 400.2542 | 8.35 | S-Adenosylmethionine | HMDB01185 | 2.33 | 0.002 | 2.27 | Amino acid | Cysteine and methionine metabolism | 0.70 | 0.001 |
| 17 | 261.0265 | 2.49 | 5-Methylthioribose 1-phosphate | HMDB00963 | 2.18 | 0.029 | 1.42 | Amino acid | Cysteine and methionine metabolism | 0.53 | 0.025 |
| 18 | 202.0560 | 0.79 | Cysteine-S-sulfate | HMDB00731 | 1.11 | 0.023 | 0.79 | Amino acid | Cysteine and methionine metabolism | 0.25 | 0.316 |
| 19 | 295.2055 | 12.78 | Glutamylphenylalanine | HMDB00594 | 1.17 | 0.015 | 1.01 | Amino acid | Glutamate metabolism | 0.62 | 0.006 |
| 20 | 232.0029 | 5.68 | Isovalerylglutamic acid | HMDB00726 | 1.09 | 0.006 | 0.94 | Amino acid | Glutamate metabolism | 0.56 | 0.016 |
| 21 | 293.0543 | 2.49 | Phenylbutyrylglutamine | HMDB11687 | 2.34 | 0.008 | 1.78 | Amino acid | Glutamine metabolism | 0.35 | 0.154 |
| 22 | 258.9796 | 2.49 | Imidazoleacetic acid riboside | HMDB02331 | 2.20 | 0.000 | 1.29 | Amino acid | Histidine metabolism | 0.49 | 0.041 |
| 23 | 170.0561 | 14.79 | 1-Methylhistidine | HMDB00001 | 1.55 | 0.035 | -2.94 | Amino acid | Histidine metabolism | -0.09 | 0.735 |
| 24 | 288.0654 | 13.04 | N-Ribosylhistidine | HMDB02089 | 1.12 | 0.029 | 0.23 | Amino acid | Histidine metabolism | 0.23 | 0.359 |
| 25 | 129.9797 | 21.87 | Pipecolic acid | HMDB00070 | 1.93 | 0.004 | 1.41 | Amino acid | Lysine metabolism | 0.25 | 0.310 |
| 26 | 172.0063 | 6.64 | Tetrahydrodipicolinate | HMDB12289 | 1.47 | 0.043 | 1.10 | Amino acid | Lysine metabolism | 0.52 | 0.029 |
| 27 | 146.0812 | 2.04 | 3-Dehydroxycarnitine | HMDB06831 | 1.30 | 0.035 | -0.59 | Amino acid | Lysine metabolism | 0.18 | 0.482 |
| 28 | 167.0228 | 6.50 | L-3-Phenyllactic acid | HMDB00748 | 2.37 | 0.010 | -2.27 | Amino acid | Phenylalanine metabolism | -0.31 | 0.206 |
| 29 | 284.1702 | 1.34 | N-Phenylacetylphenylalanine | HMDB02372 | 1.60 | 0.023 | 0.70 | Amino acid | Phenylalanine metabolism | 0.33 | 0.177 |
| 30 | 279.0749 | 2.49 | L-phenylalanyl-L-hydroxyproline | HMDB11176 | 1.60 | 0.012 | 1.23 | Amino acid | Phenylalanine metabolism | 0.32 | 0.190 |
| 31 | 208.0393 | 5.67 | N-Acetyl-L-phenylalanine | HMDB00512 | 1.46 | 0.019 | 0.87 | Amino acid | Phenylalanine metabolism | 0.61 | 0.008 |
| 32 | 166.0501 | 4.91 | L-Phenylalanine | HMDB00159 | 1.33 | 0.043 | 1.75 | Amino acid | Phenylalanine metabolism | 0.08 | 0.744 |
| 33 | 265.0863 | 6.91 | Alpha-N-Phenylacetyl-L-glutamine | HMDB06344 | 1.26 | 0.019 | 0.62 | Amino acid | Phenylalanine metabolism | 0.29 | 0.242 |
| 34 | 274.1873 | 1.83 | L-Thyronine | HMDB00667 | 1.09 | 0.043 | 0.64 | Amino acid | Phenylalanine metabolism | 0.36 | 0.143 |
| 35 | 250.0135 | 4.56 | Norepinephrine sulfate | HMDB02062 | 1.65 | 0.005 | 1.48 | Amino acid | Phenylalanine, tyrosine and tryptophan metabolism | 0.63 | 0.006 |
| 36 | 140.0412 | 2.48 | 3,4-Dihydroxybenzylamine | HMDB12153 | 1.27 | 0.043 | -0.78 | Amino acid | Phenylalanine, tyrosine and tryptophan metabolism | 0.23 | 0.363 |
| 37 | 251.0269 | 1.77 | 3,4-Dihydroxyphenylglycol O-sulfate | HMDB01474 | 1.09 | 0.029 | -0.58 | Amino acid | Phenylalanine, tyrosine and tryptophan metabolism | 0.03 | 0.899 |
| 38 | 229.0383 | 2.49 | L-leucyl-L-proline | HMDB11175 | 2.75 | 0.003 | 2.37 | Amino acid | Proline metabolism | 0.38 | 0.119 |
| 39 | 263.0218 | 2.49 | L-phenylalanyl-L-proline | HMDB11177 | 2.53 | 0.015 | 1.78 | Amino acid | Proline metabolism | 0.42 | 0.085 |
| 40 | 229.0031 | 1.97 | Prolylhydroxyproline | HMDB06695 | 2.21 | 0.010 | 2.04 | Amino acid | Proline metabolism | 0.43 | 0.072 |
| 41 | 188.9684 | 1.52 | L-glycyl-L-hydroxyproline | HMDB11173 | 1.43 | 0.010 | -1.41 | Amino acid | Proline metabolism | -0.61 | 0.008 |
| 42 | 448.0492 | 1.45 | Se-Adenosylselenomethionine | HMDB11118 | 1.71 | 0.010 | 1.22 | Amino acid | Selenocompound metabolism | 0.44 | 0.068 |
| 43 | 313.0357 | 0.86 | Gamma-Glutamyl-Se-methylselenocysteine | HMDB10716 | 1.39 | 0.005 | 1.48 | Amino acid | Selenocompound metabolism | 0.68 | 0.002 |
| 44 | 233.0600 | 2.49 | Melatonin | HMDB01389 | 2.61 | 0.005 | 1.94 | Amino acid | Tryptophan metabolism | 0.37 | 0.135 |
| 45 | 237.0057 | 2.49 | N'-Formylkynurenine | HMDB01200 | 2.03 | 0.015 | 1.54 | Amino acid | Tryptophan metabolism | 0.25 | 0.314 |
| 46 | 245.1495 | 8.54 | N-(3-(1-Indol-3-yl)-1-oxo-2-propenyl)glycine | HMDB06005 | 1.69 | 0.015 | 1.08 | Amino acid | Tryptophan metabolism | 0.59 | 0.010 |
| 47 | 249.0282 | 1.28 | 6-Hydroxymelatonin | HMDB04081 | 1.58 | 0.029 | 2.11 | Amino acid | Tryptophan metabolism | 0.33 | 0.178 |
| 48 | 191.0401 | 2.49 | 5-Methoxytryptamine | HMDB04095 | 1.55 | 0.006 | 0.66 | Amino acid | Tryptophan metabolism | 0.41 | 0.089 |
| 49 | 208.0209 | 2.49 | 4-(2-Aminophenyl)-2,4-dioxobutanoic acid | HMDB00978 | 1.43 | 0.012 | 0.67 | Amino acid | Tryptophan metabolism | 0.33 | 0.175 |
| 50 | 301.1687 | 16.68 | Cinnavalininate | HMDB04078 | 1.41 | 0.029 | -1.53 | Amino acid | Tryptophan metabolism | 0.28 | 0.262 |
| 51 | 206.1175 | 13.47 | 5-Methoxyindoleacetate | HMDB04096 | 1.38 | 0.029 | 0.56 | Amino acid | Tryptophan metabolism | 0.36 | 0.140 |
| 52 | 221.0784 | 10.02 | 5-Hydroxy-L-tryptophan | HMDB00472 | 1.38 | 0.019 | 0.97 | Amino acid | Tryptophan metabolism | 0.45 | 0.064 |
| 53 | 191.0401 | 3.02 | N-Methylserotonin | HMDB04369 | 1.37 | 0.029 | 0.94 | Amino acid | Tryptophan metabolism | 0.41 | 0.094 |
| 54 | 190.0824 | 1.70 | 3-Indolepropionic acid | HMDB02302 | 1.35 | 0.023 | -0.68 | Amino acid | Tryptophan metabolism | 0.27 | 0.270 |
| 55 | 249.0734 | 12.40 | 5-Hydroxyindoleacetylglycine | HMDB04185 | 1.34 | 0.015 | 0.90 | Amino acid | Tryptophan metabolism | 0.32 | 0.197 |
| 56 | 173.9849 | 21.92 | Quinaldic acid | HMDB00842 | 1.19 | 0.008 | 0.25 | Amino acid | Tryptophan metabolism | 0.29 | 0.237 |
| 57 | 235.1329 | 14.47 | 5-Methoxytryptophan | HMDB02339 | 1.19 | 0.029 | 1.33 | Amino acid | Tryptophan metabolism | 0.26 | 0.299 |
| 58 | 205.0287 | 1.51 | L-Tryptophan | HMDB00929 | 1.12 | 0.005 | -0.68 | Amino acid | Tryptophan metabolism | -0.59 | 0.010 |
| 59 | 219.0177 | 1.79 | N-Acetylserotonin | HMDB01238 | 1.09 | 0.001 | -0.69 | Amino acid | Tryptophan metabolism | -0.13 | 0.608 |
| 60 | 162.0761 | 1.04 | Tryptophanol | HMDB03447 | 1.02 | 0.010 | 0.76 | Amino acid | Tryptophan metabolism | 0.47 | 0.050 |
| 61 | 265.0216 | 2.49 | 3-Methoxy-4-Hydroxyphenylglycol sulfate | HMDB03332 | 2.16 | 0.006 | 2.15 | Amino acid | Tyrosine metabolism | 0.31 | 0.203 |
| 62 | 262.0135 | 3.02 | O-Phosphotyrosine | HMDB06049 | 2.04 | 0.012 | 2.02 | Amino acid | Tyrosine metabolism | 0.56 | 0.017 |
| 63 | 153.0432 | 21.76 | p-Hydroxyphenylacetic acid | HMDB00020 | 1.87 | 0.019 | 1.95 | Amino acid | Tyrosine metabolism | 0.22 | 0.389 |
| 64 | 227.0915 | 12.44 | 3-Nitrotyrosine | HMDB01904 | 1.85 | 0.008 | 0.83 | Amino acid | Tyrosine metabolism | 0.29 | 0.240 |
| 65 | 213.1121 | 10.41 | Vanillactic acid | HMDB00913 | 1.75 | 0.010 | 1.51 | Amino acid | Tyrosine metabolism | 0.37 | 0.133 |
| 66 | 211.0105 | 2.49 | Vanilpyruvic acid | HMDB11714 | 1.55 | 0.023 | 0.76 | Amino acid | Tyrosine metabolism | 0.36 | 0.144 |
| 67 | 234.0186 | 3.04 | Dopamine 3-O-sulfate | HMDB06275 | 1.55 | 0.043 | 1.60 | Amino acid | Tyrosine metabolism | 0.45 | 0.063 |
| 68 | 183.0990 | 12.18 | Homovanillic acid | HMDB00118 | 1.53 | 0.010 | 0.65 | Amino acid | Tyrosine metabolism | 0.31 | 0.218 |
| 69 | 185.0399 | 3.03 | 3,4-Dihydroxymandelic acid | HMDB01866 | 1.35 | 0.023 | 1.30 | Amino acid | Tyrosine metabolism | 0.41 | 0.091 |
| 70 | 164.9844 | 17.55 | 4-Hydroxycinnamic acid | HMDB02035 | 1.21 | 0.012 | -1.44 | Amino acid | Tyrosine metabolism | -0.41 | 0.087 |
| 71 | 311.1164 | 14.16 | Gamma-Glutamyltyrosine | HMDB11741 | 1.18 | 0.008 | 0.61 | Amino acid | Tyrosine metabolism | 0.02 | 0.942 |
| 72 | 233.9821 | 1.51 | Dopamine 4-sulfate | HMDB04148 | 1.15 | 0.005 | -0.97 | Amino acid | Tyrosine metabolism | -0.59 | 0.009 |
| 73 | 292.1235 | 15.60 | S-(3-Methylbutanoyl)-dihydrolipoamide-E | HMDB06867 | 1.26 | 0.015 | 0.99 | Amino acid | Valine, Leucine and Isoleucine Degradation | 0.39 | 0.112 |
| 74 | 277.1248 | 16.97 | Stearidonic acid | HMDB06547 | 1.63 | 0.004 | -0.99 | Lipid | alpha-Linolenic acid metabolism | 0.09 | 0.733 |
| 75 | 440.1771 | 9.40 | Leukotriene E4 | HMDB02200 | 2.14 | 0.003 | 0.89 | Lipid | Arachidonic acid metabolism | 0.59 | 0.010 |
| 76 | 317.1568 | 7.10 | 15-Deoxy-d-12,14-PGJ2 | HMDB05079 | 1.76 | 0.029 | 1.95 | Lipid | Arachidonic acid metabolism | 0.46 | 0.054 |
| 77 | 353.1859 | 13.69 | Lipoxin A4 | HMDB04385 | 1.47 | 0.019 | 0.94 | Lipid | Arachidonic acid metabolism | 0.29 | 0.248 |
| 78 | 362.2043 | 12.48 | N-Arachidonoyl glycine | HMDB05096 | 1.39 | 0.008 | 0.67 | Lipid | Arachidonic acid metabolism | 0.68 | 0.002 |
| 79 | 321.2725 | 18.21 | 11,12-EpETrE | HMDB10409 | 1.30 | 0.012 | -0.95 | Lipid | Arachidonic acid metabolism | -0.38 | 0.122 |
| 80 | 337.1467 | 17.51 | Leukotriene B4 | HMDB01085 | 1.21 | 0.035 | 0.28 | Lipid | Arachidonic acid metabolism | 0.16 | 0.532 |
| 81 | 337.1821 | 18.08 | 8-iso-PGA1 | HMDB02236 | 1.17 | 0.015 | -0.95 | Lipid | Arachidonic acid metabolism | -0.08 | 0.762 |
| 82 | 405.2096 | 8.34 | 7a,12a-Dihydroxy-3-oxo-4-cholenoic acid | HMDB00447 | 2.30 | 0.005 | 1.67 | Lipid | Bile acid metabolism | 0.57 | 0.013 |
| 83 | 409.2349 | 16.77 | 3a,4b,7a-Trihydroxy-5b-cholanoic acid | HMDB00320 | 1.18 | 0.035 | 0.35 | Lipid | Bile acid metabolism | 0.42 | 0.086 |
| 84 | 387.2781 | 18.49 | 12a-Hydroxy-3-oxocholadienic acid | HMDB00385 | 1.04 | 0.006 | 0.73 | Lipid | Bile acid metabolism | 0.32 | 0.198 |
| 85 | 357.1415 | 5.00 | Tetracosahexaenoic acid | HMDB02007 | 1.86 | 0.035 | 0.60 | Lipid | Fatty acid metabolism | 0.24 | 0.327 |
| 86 | 363.2165 | 12.89 | 19,20-DiHDPA | HMDB10214 | 1.64 | 0.023 | 0.98 | Lipid | Fatty acid metabolism | 0.66 | 0.003 |
| 87 | 430.1670 | 9.21 | Hexadecanedioic acid mono-L-carnitine ester | HMDB00712 | 2.17 | 0.005 | 1.35 | Lipid | Fatty acyl metabolism | 0.63 | 0.005 |
| 88 | 444.2805 | 8.86 | 12-Hydroxy-12-octadecanoylcarnitine | HMDB13154 | 2.01 | 0.003 | 1.83 | Lipid | Fatty acyl metabolism | 0.60 | 0.008 |
| 89 | 340.3211 | 16.03 | Docosanamide | HMDB00583 | 1.98 | 0.029 | -0.77 | Lipid | Fatty acyl metabolism | -0.05 | 0.853 |
| 90 | 428.1614 | 9.21 | Stearoylcarnitine | HMDB00848 | 1.91 | 0.001 | 0.86 | Lipid | Fatty acyl metabolism | 0.55 | 0.017 |
| 91 | 386.2205 | 12.18 | 3-Hydroxy-cis-5-tetradecenoylcarnitine | HMDB13330 | 1.39 | 0.029 | 0.38 | Lipid | Fatty acyl metabolism | 0.53 | 0.024 |
| 92 | 482.1973 | 10.07 | N-Acetyl-leukotriene E4 | HMDB05084 | 1.37 | 0.015 | 1.10 | Lipid | Fatty acyl metabolism | 0.63 | 0.005 |
| 93 | 442.3938 | 19.11 | 3-Hydroxy-11Z-octadecenoylcarnitine | HMDB13339 | 1.32 | 0.023 | -0.88 | Lipid | Fatty acyl metabolism | 0.13 | 0.600 |
| 94 | 396.3471 | 17.96 | 9,12-Hexadecadienoylcarnitine | HMDB13334 | 1.16 | 0.010 | 0.75 | Lipid | Fatty acyl metabolism | 0.61 | 0.007 |
| 95 | 442.1713 | 1.46 | Leukotriene E3 | HMDB02355 | 1.09 | 0.012 | 0.45 | Lipid | Fatty acyl metabolism | 0.45 | 0.062 |
| 96 | 351.2641 | 12.23 | 15-Keto-prostaglandin E2 | HMDB03175 | 1.07 | 0.010 | 1.37 | Lipid | Fatty acyl metabolism | 0.35 | 0.158 |
| 97 | 695.5723 | 21.09 | Diglyceride(20:1/22:6) | HMDB07411 | 2.24 | 0.023 | 1.40 | Lipid | Glycerolipid metabolism | 0.21 | 0.398 |
| 98 | 877.7267 | 20.91 | Triglyceride(18:2/16:0/20:5) | HMDB10475 | 2.16 | 0.023 | 2.15 | Lipid | Glycerolipid metabolism | -0.01 | 0.979 |
| 99 | 621.5336 | 18.10 | Diglyceride(14:0/22:2) | HMDB07030 | 2.14 | 0.023 | -1.74 | Lipid | Glycerolipid metabolism | -0.40 | 0.096 |
| 100 | 635.2993 | 10.40 | Diglyceride(18:4/20:5) | HMDB07346 | 2.11 | 0.002 | 0.93 | Lipid | Glycerolipid metabolism | 0.61 | 0.007 |
| 101 | 431.2180 | 9.73 | Monoglyceride(24:6) | HMDB11560 | 1.99 | 0.005 | 0.82 | Lipid | Glycerolipid metabolism | 0.49 | 0.039 |
| 102 | 303.2317 | 16.70 | Monoglyceride(14:0) | HMDB11530 | 1.96 | 0.035 | -2.70 | Lipid | Glycerolipid metabolism | 0.16 | 0.527 |
| 103 | 329.2109 | 12.83 | Monoglyceride(16:1) | HMDB11534 | 1.94 | 0.004 | 1.77 | Lipid | Glycerolipid metabolism | 0.56 | 0.015 |
| 104 | 851.6719 | 21.82 | Triglyceride(16:1/16:1/20:4) | HMDB05436 | 1.83 | 0.005 | -0.57 | Lipid | Glycerolipid metabolism | 0.49 | 0.037 |
| 105 | 841.6504 | 21.49 | Triglyceride(15:0/18:1/18:3) | HMDB11709 | 1.78 | 0.015 | -1.08 | Lipid | Glycerolipid metabolism | 0.19 | 0.452 |
| 106 | 466.3893 | 18.05 | Lysosphingomyelin(d18:1) | HMDB06482 | 1.66 | 0.035 | -0.97 | Lipid | Glycerolipid metabolism | 0.10 | 0.706 |
| 107 | 901.6372 | 21.36 | Triglyceride(18:2/18:2/20:5) | HMDB10493 | 1.54 | 0.023 | 0.94 | Lipid | Glycerolipid metabolism | -0.06 | 0.810 |
| 108 | 825.6851 | 21.59 | Triglyceride(18:2/14:0/18:3) | HMDB10471 | 1.52 | 0.010 | -0.84 | Lipid | Glycerolipid metabolism | 0.25 | 0.315 |
| 109 | 827.6907 | 21.64 | Triglyceride(16:1/16:0/18:3) | HMDB10424 | 1.47 | 0.035 | -0.64 | Lipid | Glycerolipid metabolism | 0.13 | 0.601 |
| 110 | 705.5255 | 19.29 | Diglyceride(18:2/24:0) | HMDB07267 | 1.46 | 0.043 | -0.98 | Lipid | Glycerolipid metabolism | 0.25 | 0.321 |
| 111 | 343.2842 | 16.91 | Monoglyceride(P-18:0e) | HMDB11153 | 1.44 | 0.035 | 1.76 | Lipid | Glycerolipid metabolism | 0.49 | 0.041 |
| 112 | 717.5760 | 21.55 | Diglyceride(22:4/22:6) | HMDB07701 | 1.32 | 0.035 | -1.49 | Lipid | Glycerolipid metabolism | 0.03 | 0.914 |
| 113 | 717.5457 | 20.34 | Diglyceride(22:4/22:5) | HMDB07699 | 1.30 | 0.023 | 0.56 | Lipid | Glycerolipid metabolism | 0.28 | 0.255 |
| 114 | 381.1357 | 15.07 | Monoglyceride(20:3) | HMDB11577 | 1.24 | 0.012 | 1.08 | Lipid | Glycerolipid metabolism | 0.44 | 0.071 |
| 115 | 733.5565 | 19.80 | Diglyceride(20:2/24:0) | HMDB07441 | 1.19 | 0.035 | -0.52 | Lipid | Glycerolipid metabolism | 0.29 | 0.241 |
| 116 | 823.6398 | 20.99 | Triglyceride(18:3/14:0/18:3) | HMDB10497 | 1.14 | 0.023 | -0.79 | Lipid | Glycerolipid metabolism | 0.01 | 0.978 |
| 117 | 411.1941 | 11.51 | Monoglyceride(22:2) | HMDB11553 | 1.13 | 0.029 | -0.32 | Lipid | Glycerolipid metabolism | 0.23 | 0.353 |
| 118 | 775.5465 | 20.12 | Triglyceride(16:1/14:0/16:1) | HMDB10419 | 1.08 | 0.010 | 0.70 | Lipid | Glycerolipid metabolism | 0.32 | 0.202 |
| 119 | 725.5551 | 19.87 | Diglyceride(20:5/24:1) | HMDB07587 | 1.06 | 0.035 | 0.68 | Lipid | Glycerolipid metabolism | -0.17 | 0.496 |
| 120 | 383.2919 | 18.76 | Monoglyceride(20:2) | HMDB11544 | 1.02 | 0.029 | 1.16 | Lipid | Glycerolipid metabolism | 0.24 | 0.346 |
| 121 | 504.2128 | 9.71 | Lysophosphatidylethanolamine(20:3) | HMDB11484 | 2.68 | 0.000 | 1.68 | Lipid | Glycerophospholipid metabolism | 0.70 | 0.001 |
| 122 | 489.2235 | 9.71 | Citicoline | HMDB01413 | 2.60 | 0.001 | 1.46 | Lipid | Glycerophospholipid metabolism | 0.66 | 0.003 |
| 123 | 454.2995 | 21.85 | Lysophosphatidylethanolamine(16:0) | HMDB11473 | 2.56 | 0.023 | -2.22 | Lipid | Glycerophospholipid metabolism | -0.14 | 0.578 |
| 124 | 566.2283 | 10.37 | Lysophosphatidylethanolamine(24:0) | HMDB11497 | 2.41 | 0.010 | 2.18 | Lipid | Glycerophospholipid metabolism | 0.65 | 0.004 |
| 125 | 520.5127 | 9.71 | Lysophosphatidylcholine(18:2) | HMDB10386 | 2.28 | 0.001 | 1.05 | Lipid | Glycerophospholipid metabolism | 0.65 | 0.003 |
| 126 | 850.6688 | 21.82 | Phosphatidylethanolamine(20:4/24:1) | HMDB09410 | 2.22 | 0.004 | -0.70 | Lipid | Glycerophospholipid metabolism | 0.35 | 0.150 |
| 127 | 818.5664 | 20.18 | Phosphatidylethanolamine(20:1/22:6) | HMDB09276 | 2.09 | 0.008 | -2.55 | Lipid | Glycerophospholipid metabolism | 0.01 | 0.972 |
| 128 | 834.6367 | 18.97 | Phosphatidylcholine(18:1/22:5) | HMDB08088 | 2.08 | 0.029 | -1.71 | Lipid | Glycerophospholipid metabolism | -0.52 | 0.028 |
| 129 | 452.2765 | 17.02 | Lysophosphatidylethanolamine(16:1) | HMDB11474 | 2.07 | 0.002 | -1.27 | Lipid | Glycerophospholipid metabolism | 0.19 | 0.461 |
| 130 | 435.1814 | 10.40 | Lysophoaphatidic acid(18:2) | HMDB07852 | 2.06 | 0.000 | 1.40 | Lipid | Glycerophospholipid metabolism | 0.67 | 0.002 |
| 131 | 824.6082 | 21.63 | Phosphatidylethanolamine(18:3/24:1) | HMDB09146 | 1.95 | 0.043 | -1.22 | Lipid | Glycerophospholipid metabolism | 0.36 | 0.144 |
| 132 | 516.3021 | 16.97 | Lysophosphatidylcholine(18:4) | HMDB10389 | 1.92 | 0.035 | -0.83 | Lipid | Glycerophospholipid metabolism | -0.40 | 0.101 |
| 133 | 852.6752 | 21.82 | Phosphatidylethanolamine(20:4/24:0) | HMDB09409 | 1.83 | 0.012 | -0.56 | Lipid | Glycerophospholipid metabolism | 0.23 | 0.355 |
| 134 | 894.6357 | 20.91 | Phosphatidylcholine(20:4/24:0) | HMDB08453 | 1.77 | 0.004 | -0.41 | Lipid | Glycerophospholipid metabolism | 0.40 | 0.104 |
| 135 | 900.6835 | 21.75 | Phosphatidylcholine(20:0/24:1) | HMDB08290 | 1.76 | 0.003 | -0.86 | Lipid | Glycerophospholipid metabolism | 0.33 | 0.182 |
| 136 | 754.5358 | 19.85 | Phosphatidylcholine(14:0/20:4) | HMDB07883 | 1.73 | 0.035 | -2.82 | Lipid | Glycerophospholipid metabolism | -0.47 | 0.049 |
| 137 | 820.5830 | 19.98 | Phosphatidylethanolamine(20:0/22:6) | HMDB09243 | 1.69 | 0.023 | -2.22 | Lipid | Glycerophospholipid metabolism | -0.19 | 0.446 |
| 138 | 634.4807 | 19.19 | Phosphatidylethanolamine(14:0/14:1) | HMDB08822 | 1.68 | 0.012 | -1.05 | Lipid | Glycerophospholipid metabolism | 0.21 | 0.398 |
| 139 | 834.6705 | 21.63 | Phosphatidylcholine(18:0/22:6) | HMDB08057 | 1.66 | 0.043 | -0.34 | Lipid | Glycerophospholipid metabolism | 0.21 | 0.400 |
| 140 | 749.5826 | 20.74 | Phosphatidylglycerol(16:0/18:1) | HMDB10573 | 1.63 | 0.029 | -0.22 | Lipid | Glycerophospholipid metabolism | 0.33 | 0.183 |
| 141 | 902.6902 | 21.71 | Phosphatidylcholine(20:0/24:0) | HMDB08289 | 1.62 | 0.019 | -0.93 | Lipid | Glycerophospholipid metabolism | -0.16 | 0.532 |
| 142 | 748.6402 | 21.15 | Phosphatidylethanolamine(O-16:1/22:6) | HMDB05780 | 1.61 | 0.001 | 1.23 | Lipid | Glycerophospholipid metabolism | 0.13 | 0.609 |
| 143 | 718.5745 | 20.17 | Phosphatidylcholine(14:0/P-18:0) | HMDB07896 | 1.54 | 0.005 | -0.41 | Lipid | Glycerophospholipid metabolism | -0.07 | 0.772 |
| 144 | 800.5555 | 20.86 | Phosphatidylcholine(18:4/20:5) | HMDB08248 | 1.46 | 0.005 | -0.55 | Lipid | Glycerophospholipid metabolism | -0.39 | 0.114 |
| 145 | 880.5854 | 19.38 | Phosphatidylethanolamine(22:4/24:0) | HMDB09606 | 1.45 | 0.006 | -0.62 | Lipid | Glycerophospholipid metabolism | 0.21 | 0.402 |
| 146 | 506.3844 | 18.47 | Lysophosphatidylethanolamine(20:2) | HMDB11483 | 1.44 | 0.003 | -0.86 | Lipid | Glycerophospholipid metabolism | -0.54 | 0.020 |
| 147 | 836.6138 | 21.35 | Phosphatidylcholine(18:1/22:4) | HMDB08087 | 1.39 | 0.029 | -0.43 | Lipid | Glycerophospholipid metabolism | 0.33 | 0.181 |
| 148 | 522.3858 | 19.16 | Lysophosphatidylcholine(18:1) | HMDB10385 | 1.38 | 0.023 | -0.81 | Lipid | Glycerophospholipid metabolism | 0.35 | 0.158 |
| 149 | 538.4231 | 18.49 | Lysophosphatidylethanolamine(22:0) | HMDB11490 | 1.37 | 0.019 | -1.12 | Lipid | Glycerophospholipid metabolism | 0.30 | 0.222 |
| 150 | 776.5486 | 20.12 | Phosphatidylethanolamine(22:5/P-18:1) | HMDB09677 | 1.35 | 0.003 | 0.56 | Lipid | Glycerophospholipid metabolism | 0.41 | 0.090 |
| 151 | 838.6128 | 20.36 | Phosphatidylcholine(18:3/22:1) | HMDB08184 | 1.32 | 0.004 | -0.28 | Lipid | Glycerophospholipid metabolism | 0.20 | 0.429 |
| 152 | 726.5346 | 20.19 | Phosphatidylethanolamine(18:3/P-18:0) | HMDB09148 | 1.32 | 0.003 | 0.88 | Lipid | Glycerophospholipid metabolism | 0.40 | 0.098 |
| 153 | 846.6302 | 20.72 | Phosphatidylethanolamine(22:1/22:6) | HMDB09540 | 1.30 | 0.008 | -0.78 | Lipid | Glycerophospholipid metabolism | -0.13 | 0.596 |
| 154 | 578.2776 | 16.98 | Lysophosphatidylcholine(22:1) | HMDB10399 | 1.30 | 0.003 | -0.84 | Lipid | Glycerophospholipid metabolism | 0.18 | 0.478 |
| 155 | 724.5248 | 20.80 | Phosphatidylethanolamine(18:3/P-18:1) | HMDB09150 | 1.28 | 0.015 | 0.71 | Lipid | Glycerophospholipid metabolism | 0.53 | 0.024 |
| 156 | 572.3619 | 17.26 | Lysophosphatidylcholine(22:4) | HMDB10401 | 1.27 | 0.035 | -0.71 | Lipid | Glycerophospholipid metabolism | 0.47 | 0.051 |
| 157 | 798.5406 | 20.70 | Phosphatidylethanolamine(18:3/22:0) | HMDB09138 | 1.23 | 0.002 | 0.71 | Lipid | Glycerophospholipid metabolism | 0.11 | 0.655 |
| 158 | 894.5984 | 19.30 | Phosphatidylcholine(20:3/24:1) | HMDB08389 | 1.22 | 0.008 | -1.18 | Lipid | Glycerophospholipid metabolism | 0.35 | 0.158 |
| 159 | 868.5838 | 19.11 | Phosphatidylcholine(18:3/24:0) | HMDB08190 | 1.21 | 0.035 | -0.78 | Lipid | Glycerophospholipid metabolism | 0.53 | 0.024 |
| 160 | 564.4384 | 18.53 | Lysophosphatidylethanolamine(24:1) | HMDB11498 | 1.19 | 0.035 | -0.39 | Lipid | Glycerophospholipid metabolism | -0.26 | 0.296 |
| 161 | 750.5337 | 20.05 | Phosphatidylethanolamine(20:4/P-18:1) | HMDB09446 | 1.16 | 0.012 | 0.73 | Lipid | Glycerophospholipid metabolism | 0.34 | 0.166 |
| 162 | 856.5746 | 19.35 | Phosphatidylcholine(20:3/22:6) | HMDB08419 | 1.16 | 0.029 | -0.42 | Lipid | Glycerophospholipid metabolism | 0.16 | 0.514 |
| 163 | 768.5512 | 21.24 | Phosphatidylcholine(18:3/P-18:0) | HMDB08193 | 1.10 | 0.003 | 0.91 | Lipid | Glycerophospholipid metabolism | 0.41 | 0.091 |
| 164 | 771.5668 | 19.80 | Phosphatidylglycerol(16:0/20:4) | HMDB10580 | 1.07 | 0.029 | -0.40 | Lipid | Glycerophospholipid metabolism | 0.20 | 0.421 |
| 165 | 848.6137 | 20.71 | Phosphatidylethanolamine(20:5/24:1) | HMDB09476 | 1.05 | 0.010 | -0.59 | Lipid | Glycerophospholipid metabolism | 0.22 | 0.385 |
| 166 | 819.6089 | 20.20 | Phosphatidylglycerol(18:2/22:6) | HMDB10659 | 1.04 | 0.012 | -0.60 | Lipid | Glycerophospholipid metabolism | 0.11 | 0.673 |
| 167 | 297.0385 | 7.63 | 13S-hydroxyoctadecadienoic acid | HMDB04667 | 2.88 | 0.008 | -2.65 | Lipid | Linoleic acid metabolism | 0.33 | 0.180 |
| 168 | 331.2265 | 13.24 | 9,12,13-TriHOME | HMDB04708 | 2.06 | 0.002 | 0.93 | Lipid | Linoleic acid metabolism | 0.68 | 0.002 |
| 169 | 620.5313 | 18.43 | C22:1-ceramide | HMDB11775 | 2.15 | 0.002 | -1.57 | Lipid | Sphingolipid metabolism | -0.36 | 0.148 |
| 170 | 974.7407 | 21.37 | Galabiosylceramide(d18:1/24:0) | HMDB04840 | 1.98 | 0.001 | -0.85 | Lipid | Sphingolipid metabolism | -0.15 | 0.552 |
| 171 | 787.6696 | 21.92 | Sphingomyelin(d18:0/22:1) | HMDB12092 | 1.85 | 0.035 | -1.71 | Lipid | Sphingolipid metabolism | 0.12 | 0.648 |
| 172 | 646.5461 | 18.49 | C18-ceramide phosphate | HMDB10701 | 1.72 | 0.015 | -0.59 | Lipid | Sphingolipid metabolism | -0.47 | 0.051 |
| 173 | 704.5220 | 19.29 | Sphingomyelin(d18:1/16:0) | HMDB10169 | 1.66 | 0.035 | -0.62 | Lipid | Sphingolipid metabolism | 0.09 | 0.717 |
| 174 | 756.5906 | 21.75 | Glucosylceramide(d18:1/20:0) | HMDB04973 | 1.66 | 0.023 | -1.51 | Lipid | Sphingolipid metabolism | 0.53 | 0.024 |
| 175 | 838.6207 | 21.36 | Glucosylceramide(d18:1/26:1) | HMDB04976 | 1.44 | 0.029 | -0.36 | Lipid | Sphingolipid metabolism | 0.09 | 0.736 |
| 176 | 810.6817 | 21.62 | Glucosylceramide(d18:1/24:1) | HMDB04975 | 1.41 | 0.008 | -0.40 | Lipid | Sphingolipid metabolism | 0.29 | 0.243 |
| 177 | 972.7344 | 21.37 | Galabiosylceramide(d18:1/24:1) | HMDB04837 | 1.23 | 0.008 | -0.50 | Lipid | Sphingolipid metabolism | 0.46 | 0.055 |
| 178 | 761.6537 | 21.44 | Sphingomyelin(d18:0/20:0) | HMDB12090 | 1.14 | 0.002 | 0.73 | Lipid | Sphingolipid metabolism | 0.53 | 0.024 |
| 179 | 361.1832 | 7.77 | Aldosterone | HMDB00037 | 2.12 | 0.008 | 2.09 | Lipid | Steroid hormone biosynthesis | 0.49 | 0.039 |
| 180 | 347.2217 | 13.23 | Cortexolone | HMDB00015 | 2.03 | 0.002 | 1.34 | Lipid | Steroid hormone biosynthesis | 0.51 | 0.029 |
| 181 | 449.2357 | 8.86 | 17-beta-estradiol-3-glucuronide | HMDB06224 | 1.82 | 0.019 | 1.00 | Lipid | Steroid hormone biosynthesis | 0.61 | 0.007 |
| 182 | 285.2060 | 15.31 | 16-Oxoestrone | HMDB00372 | 1.69 | 0.000 | 1.63 | Lipid | Steroid hormone biosynthesis | 0.22 | 0.384 |
| 183 | 365.2911 | 11.26 | Dihydrocortisol | HMDB03259 | 1.29 | 0.012 | 0.91 | Lipid | Steroid hormone biosynthesis | 0.40 | 0.101 |
| 184 | 291.1254 | 16.17 | Dihydrotestosterone | HMDB02961 | 1.22 | 0.043 | 1.31 | Lipid | Steroid hormone biosynthesis | 0.43 | 0.075 |
| 185 | 365.1782 | 17.92 | 11b,21-Dihydroxy-3,20-oxo-5b-pregnan-18-al | HMDB06754 | 1.21 | 0.029 | 0.48 | Lipid | Steroid hormone biosynthesis | 0.03 | 0.913 |
| 186 | 273.1009 | 13.03 | Estradiol | HMDB00151 | 1.06 | 0.023 | 0.62 | Lipid | Steroid hormone biosynthesis | 0.14 | 0.589 |
| 187 | 341.2472 | 17.04 | Canrenone | HMDB03033 | 1.94 | 0.001 | 1.18 | Lipid | Steroid metabolism | 0.25 | 0.318 |
| 188 | 303.1954 | 13.35 | Enterodiol | HMDB05056 | 1.76 | 0.006 | 1.25 | Lipid | Steroid metabolism | 0.55 | 0.018 |
| 189 | 497.3285 | 16.97 | Pregnanediol-3-glucuronide | HMDB10318 | 1.61 | 0.029 | -0.71 | Lipid | Steroid metabolism | 0.34 | 0.165 |
| 190 | 383.1828 | 12.48 | Farnesyl pyrophosphate | HMDB00961 | 1.32 | 0.004 | 0.69 | Lipid | Steroid metabolism | 0.67 | 0.002 |
| 191 | 244.0871 | 2.51 | Biotin amide | HMDB01458 | 3.05 | 0.004 | 3.63 | Cofactors and vitamins | Biotin metabolism | 0.59 | 0.009 |
| 192 | 277.0769 | 2.49 | Biotin sulfone | HMDB04818 | 2.42 | 0.004 | 2.14 | Cofactors and vitamins | Biotin metabolism | 0.45 | 0.060 |
| 193 | 373.3138 | 15.95 | Biocytin | HMDB03134 | 1.90 | 0.029 | -1.20 | Cofactors and vitamins | Biotin metabolism | 0.12 | 0.642 |
| 194 | 217.1435 | 13.04 | Bisnorbiotin | HMDB04821 | 1.34 | 0.019 | 0.47 | Cofactors and vitamins | Biotin metabolism | 0.43 | 0.073 |
| 195 | 254.0696 | 2.49 | Neopterin | HMDB00845 | 2.52 | 0.008 | 2.05 | Cofactors and vitamins | Folate metabolism | 0.38 | 0.125 |
| 196 | 336.0151 | 0.90 | Dihydroneopterin phosphate | HMDB06824 | 1.68 | 0.029 | 2.47 | Cofactors and vitamins | Folate metabolism | 0.54 | 0.020 |
| 197 | 207.0176 | 2.75 | Lipoic acid | HMDB01451 | 1.55 | 0.010 | 1.36 | Cofactors and vitamins | Lipoic acid metabolism | 0.59 | 0.010 |
| 198 | 335.0915 | 6.97 | Nicotinamide ribotide | HMDB00229 | 1.10 | 0.035 | -1.09 | Cofactors and vitamins | Nicotinate and nicotinamide metabolism | 0.32 | 0.188 |
| 199 | 473.1852 | 5.90 | 5-Formiminotetrahydrofolic acid | HMDB01534 | 1.82 | 0.019 | 1.16 | Cofactors and vitamins | One carbon pool by folate | 0.34 | 0.169 |
| 200 | 301.1797 | 12.65 | Retinoic acid | HMDB01852 | 1.63 | 0.004 | 0.92 | Cofactors and vitamins | Retinol metabolism | 0.57 | 0.013 |
| 201 | 357.0856 | 15.12 | 5-Amino-6-(5'-phosphoribitylamino)uracil | HMDB03841 | 1.43 | 0.029 | 1.35 | Cofactors and vitamins | Riboflavin metabolism | 0.69 | 0.002 |
| 202 | 327.1475 | 16.19 | 6,7-Dimethyl-8-(1-D-ribityl)lumazine | HMDB03826 | 1.30 | 0.023 | 1.06 | Cofactors and vitamins | Riboflavin metabolism | 0.47 | 0.048 |
| 203 | 349.1985 | 16.08 | Riboflavin reduced | HMDB01557 | 1.23 | 0.008 | 0.48 | Cofactors and vitamins | Riboflavin metabolism | 0.55 | 0.017 |
| 204 | 345.0162 | 2.49 | Thiamine monophosphate | HMDB02666 | 2.78 | 0.002 | 1.88 | Cofactors and vitamins | Thiamine metabolism | 0.43 | 0.074 |
| 205 | 266.0628 | 2.49 | Thiamine | HMDB00235 | 2.64 | 0.003 | 1.81 | Cofactors and vitamins | Thiamine metabolism | 0.44 | 0.067 |
| 206 | 247.9648 | 14.10 | Pyridoxal 5'-phosphate | HMDB01491 | 2.40 | 0.023 | 2.81 | Cofactors and vitamins | Thiamine metabolism | 0.33 | 0.184 |
| 207 | 249.9771 | 3.02 | Pyridoxine 5'-phosphate | HMDB01319 | 1.22 | 0.015 | 1.39 | Cofactors and vitamins | Vitamin B6 metabolism | 0.49 | 0.037 |
| 208 | 417.3560 | 16.31 | 23S,25-dihydroxyvitamin D3 | HMDB06720 | 2.12 | 0.012 | -1.79 | Cofactors and vitamins | Vitamin D metabolism | 0.32 | 0.194 |
| 209 | 445.3127 | 21.82 | 1-a,24R,25-Trihydroxyvitamin D2 | HMDB06227 | 1.82 | 0.015 | -0.66 | Cofactors and vitamins | Vitamin D metabolism | -0.03 | 0.919 |
| 210 | 429.3726 | 20.07 | Ercalcitriol | HMDB06225 | 1.00 | 0.043 | 0.33 | Cofactors and vitamins | Vitamin D metabolism | -0.01 | 0.966 |
| 211 | 279.2317 | 18.18 | Alpha-CEHC | HMDB01518 | 1.04 | 0.043 | -0.39 | Cofactors and vitamins | Vitamin E metabolism | -0.21 | 0.401 |
| 212 | 283.0322 | 2.49 | 1-Methylinosine | HMDB02721 | 2.62 | 0.005 | 1.66 | Nucleotide | Purine metabolism | 0.39 | 0.111 |
| 213 | 269.0524 | 2.49 | Inosine | HMDB00195 | 2.35 | 0.043 | 1.84 | Nucleotide | Purine metabolism | 0.23 | 0.362 |
| 214 | 137.0268 | 1.50 | Hypoxanthine | HMDB00157 | 2.19 | 0.029 | 2.57 | Nucleotide | Purine metabolism | -0.11 | 0.678 |
| 215 | 269.1261 | 6.03 | Arabinosylhypoxanthine | HMDB03040 | 2.03 | 0.006 | 1.44 | Nucleotide | Purine metabolism | 0.58 | 0.012 |
| 216 | 339.2142 | 15.06 | AICAR | HMDB01517 | 1.79 | 0.002 | -0.32 | Nucleotide | Purine metabolism | -0.18 | 0.477 |
| 217 | 252.0655 | 9.15 | Deoxyadenosine | HMDB00101 | 1.74 | 0.002 | 1.04 | Nucleotide | Purine metabolism | 0.28 | 0.255 |
| 218 | 230.1135 | 1.47 | 5-Phosphoribosylamine | HMDB01128 | 1.72 | 0.008 | 2.12 | Nucleotide | Purine metabolism | 0.25 | 0.312 |
| 219 | 168.0147 | 1.99 | 2,8-Dihydroxyadenine | HMDB00401 | 1.48 | 0.035 | -0.22 | Nucleotide | Purine metabolism | -0.51 | 0.032 |
| 220 | 253.1071 | 11.87 | Deoxyinosine | HMDB00071 | 1.33 | 0.015 | 0.79 | Nucleotide | Purine metabolism | 0.53 | 0.022 |
| 221 | 312.1481 | 14.33 | N2,N2-Dimethylguanosine | HMDB04824 | 1.10 | 0.010 | -0.61 | Nucleotide | Purine metabolism | -0.10 | 0.680 |
| 222 | 169.0356 | 1.47 | Uric acid | HMDB00289 | 1.09 | 0.029 | 0.39 | Nucleotide | Purine metabolism | 0.53 | 0.025 |
| 223 | 413.0438 | 9.88 | dIDP | HMDB03536 | 1.04 | 0.010 | 0.45 | Nucleotide | Purine metabolism | 0.54 | 0.020 |
| 224 | 388.0427 | 2.49 | dCDP | HMDB01244 | 3.04 | 0.006 | 2.62 | Nucleotide | Pyrimidine metabolism | 0.30 | 0.225 |
| 225 | 309.0315 | 7.57 | dUMP | HMDB01409 | 2.95 | 0.008 | -2.95 | Nucleotide | Pyrimidine metabolism | 0.05 | 0.847 |
| 226 | 325.0039 | 7.55 | Uridine 5'-monophosphate | HMDB00288 | 2.87 | 0.035 | -3.45 | Nucleotide | Pyrimidine metabolism | 0.10 | 0.701 |
| 227 | 308.1037 | 1.28 | dCMP | HMDB01202 | 2.86 | 0.005 | 2.76 | Nucleotide | Pyrimidine metabolism | 0.56 | 0.016 |
| 228 | 305.9856 | 7.58 | Cytidine 2',3'-cyclic phosphate | HMDB11691 | 2.83 | 0.010 | -3.19 | Nucleotide | Pyrimidine metabolism | 0.33 | 0.184 |
| 229 | 244.0873 | 1.91 | Cytidine | HMDB00089 | 2.62 | 0.015 | 2.02 | Nucleotide | Pyrimidine metabolism | 0.58 | 0.012 |
| 230 | 307.0789 | 8.42 | Uridine 2',3'-cyclic phosphate | HMDB11640 | 2.44 | 0.015 | -2.14 | Nucleotide | Pyrimidine metabolism | -0.08 | 0.766 |
| 231 | 243.0839 | 1.90 | Thymidine | HMDB00273 | 2.36 | 0.012 | 2.12 | Nucleotide | Pyrimidine metabolism | 0.60 | 0.008 |
| 232 | 247.1442 | 6.05 | 5,6-Dihydrouridine | HMDB00497 | 2.29 | 0.010 | 1.72 | Nucleotide | Pyrimidine metabolism | 0.62 | 0.006 |
| 233 | 227.9748 | 2.99 | Deoxycytidine | HMDB00014 | 2.12 | 0.005 | -1.72 | Nucleotide | Pyrimidine metabolism | -0.26 | 0.293 |
| 234 | 286.1218 | 1.29 | N4-Acetylcytidine | HMDB05923 | 1.74 | 0.035 | 1.39 | Nucleotide | Pyrimidine metabolism | 0.14 | 0.574 |
| 235 | 289.1321 | 16.17 | Orotidine | HMDB00788 | 1.47 | 0.043 | 1.34 | Nucleotide | Pyrimidine metabolism | 0.43 | 0.076 |
| 236 | 245.0697 | 12.18 | Pseudouridine | HMDB00767 | 1.22 | 0.008 | 1.20 | Nucleotide | Pyrimidine metabolism | 0.36 | 0.140 |
| 237 | 229.0733 | 15.69 | Deoxyuridine | HMDB00012 | 1.08 | 0.043 | 0.72 | Nucleotide | Pyrimidine metabolism | 0.38 | 0.116 |
| 238 | 246.9774 | 1.16 | Dimethylallylpyrophosphate | HMDB01120 | 1.17 | 0.010 | -0.51 | Terpenoids and polyketides | Terpenoid backbone biosynthesis | 0.35 | 0.159 |
| 239 | 517.3054 | 16.97 | Dermatan | HMDB00632 | 1.88 | 0.029 | -0.93 | Glycan biosynthesis and metabolism | Glycosaminoglycan metabolism | -0.38 | 0.119 |
| 240 | 336.2280 | 13.39 | Aspartylglycosamine | HMDB00489 | 1.38 | 0.019 | -0.65 | Glycan biosynthesis and metabolism | Glycosaminoglycan metabolism | -0.30 | 0.232 |

Metabolites were identified on the basis of accurate mass data, retention time, experimental MS/MS spectra, and library MS/MS spectra (HMDB, MyCompoundID). A total of 240 metabolites were selected among the meaningful metabolites (*n* = 588, VIP > 1.0, p < 0.005) after excluding for drugs (*n* = 20), xenobiotics (*n* = 82), differential metabolites at week 0 (*n* = 147), and a metabolite identification fit score under 0.9 (*n* = 99). A total of 240 metabolites are listed in the order of VIP scores within each pathway group. Fold change was calculated by dividing the value of metabolite intensity in week 2 of the LCKD by that in week 2 of the GD. Pearson’s *r* correlation analysis was performed to show the association between changes in β-hydroxybutyrate and each metabolite at week 2. P-value < 0.05 represents significant differences between GD and LCKD at week 2, as determined by Mann-Whitney U test.

VIP, Variable Importance in Projection; FC, fold change

**Supplementary Table S3. Metabolic pathway analysis with 65 metabolites**

| **No.** | **Pathway name** | **FDR** | **Impact** | **Match status** | **Hit metabolite** | **Log_10_FC** | ***p*-value** |
| --- | --- | --- | --- | --- | --- | --- | --- |
| 1 | Sphingolipid metabolism | 0.003 | 0.337 | 3/25 | C22:1-Ceramide  Sphingomyelin(d18:0/20:0)  Galabiosylceramide(d18:1/24:0) | -1.57  0.73  -0.85 | 0.002  0.002  0.001 |
| 2 | Tryptophan metabolism | 0.004 | 0.137 | 3/79 | L-Tryptophan  Melatonin  N-Acetylserotonin | -0.11  1.64  -0.29 | 0.005  0.005  0.001 |
| 3 | Ascorbate and aldarate metabolism | 0.007 | 0.108 | 2/45 | L-Gulonolactone  Diketogulonic acid | 0.49  0.59 | 0.004  0.002 |
| 4 | Glycerophospholipid metabolism | 0.005 | 0.375 | 5/39 | Phosphatidylcholine(14:0/P-18:0)  Phosphatidylcholine(18:3/22:1)  Phosphatidylcholine(18:3/P-18:0)  Phosphatidylcholine(18:4/20:5)  Phosphatidylcholine(20:0/24:1)  Phosphatidylcholine(20:4/24:0)  Phosphatidylethanolamine(18:3/22:0)  Phosphatidylethanolamine(18:3/P-18:0)  Phosphatidylethanolamine(20:4/24:1)  Phosphatidylethanolamine(22:5/P-18:1)  Phosphatidylethanolamine(O-16:1/22:6)  Lysophosphatidylcholine(18:2)  Lysophosphatidylcholine(22:1)  Lysophosphatidic acid(18:2)  Citicoline | -0.41  -0.28  0.91  -0.55  -0.86  -0.41  0.71  0.88  -0.70  0.56  1.23  1.05  -0.84  1.40  1.46 | 0.005  0.004  0.003  0.005  0.003  0.004  0.002  0.003  0.004  0.003  0.001  0.001  0.003  <0.001  0.001 |
| 5 | Thiamine metabolism | 0.011 | 0.294 | 2/24 | Thiamine  Thiamine monophosphate | 1.72  1.57 | 0.003  0.002 |
| 6 | Cysteine and methionine metabolism | 0.011 | 0.108 | 2/56 | S-Adenosylmethionine  S-Adenosylmethioninamine | 1.73  1.85 | 0.002  0.002 |
| 7 | Retinol metabolism | 0.015 | 0.175 | 1/22 | Retinoic acid | 0.75 | 0.004 |
| 8 | Starch and sucrose metabolism | 0.040 | 0.150 | 2/50 | Glucose 1-phosphate  Octanoylglucuronide | 1.10  2.10 | 0.004  0.005 |

Metabolic pathway analysis was performed in MetaboAnalyst 4.0 using 65 metabolites (VIP > 1.0, p < 0.005) and cut-off values was FDR-adjusted < 0.05. Match status represents the number (A) of metabolites in the metabolic pathway and the number (B) of hit metabolites analysed in this study, and displayed in the form of B/A. Especially, the 5 hit metabolites in glycerophospholipid metabolism, which do not take into account fatty acid composition, means 5 of phosphatidylcholine, phosphatidylethanolamine, lysophosphatidylcholine, lysophosphatidic acid, citicoline. Hit metabolites indicates the matched metabolites data uploaded in MetaboAnalyst 4.0. The pathway impact score was calculated from pathway topology analysis. Fold change was calculated by dividing the value of metabolite intensity in the LCKD week 2 by that in the GD week 2. p < 0.05 represents significant differences between the GD and LCKD at week 2, as determined by Mann-Whitney U test. FDR, false discovery rate; FC, fold change

**Supplementary Table S4. Correlation analysis between metabolites significantly altered at week 2 in LCKD group and β-hydroxybutyrate changes.**

| **No.** | **m/z** | **RT**  **(min)** | **Metabolite** | **HMDB** | **Correlation** | | **VIP** | ***p*-value** | **Log_10_FC** | **Superpathway** | **Pathway** |
| --- | --- | --- | --- | --- | --- | --- | --- | --- | --- | --- | --- |
|  |  |  |  |  | **r** | ***p*** |  |  |  |  |  |
| 1 | 504.2128 | 9.71 | Lysophosphatidylethanolamine(20:3) | HMDB11484 | 0.70 | 0.001 | 2.68 | 0.000 | 1.68 | Lipid | Glycerophospholipid metabolism |
| 2 | 400.2542 | 8.35 | S-Adenosylmethionine | HMDB01185 | 0.70 | 0.001 | 2.33 | 0.002 | 2.27 | Amino acid | Cysteine and methionine metabolism |
| 3 | 357.0856 | 15.12 | 5-Amino-6-(5'-phosphoribitylamino)uracil | HMDB03841 | 0.69 | 0.002 | 1.43 | 0.029 | 1.35 | Cofactors and vitamins | Riboflavin metabolism |
| 4 | 362.2043 | 12.48 | N-Arachidonoyl glycine | HMDB05096 | 0.68 | 0.002 | 1.39 | 0.008 | 0.67 | Lipid | Arachidonic acid metabolism |
| 5 | 313.0357 | 0.86 | Gamma-Glutamyl-Se-methylselenocysteine | HMDB10716 | 0.68 | 0.002 | 1.39 | 0.005 | 1.48 | Amino acid | Selenocompound metabolism |
| 6 | 331.2265 | 13.24 | 9,12,13-TriHOME | HMDB04708 | 0.68 | 0.002 | 2.06 | 0.002 | 0.93 | Lipid | Linoleic acid metabolism |
| 7 | 435.1814 | 10.40 | Lysophoaphatidic acid(18:2) | HMDB07852 | 0.67 | 0.002 | 2.06 | 0.000 | 1.40 | Lipid | Glycerophospholipid metabolism |
| 8 | 383.1828 | 12.48 | Farnesyl pyrophosphate | HMDB00961 | 0.67 | 0.002 | 1.32 | 0.004 | 0.69 | Lipid | Steroid metabolism |
| 9 | 363.2165 | 12.89 | 19,20-DiHDPA | HMDB10214 | 0.66 | 0.003 | 1.64 | 0.023 | 0.98 | Lipid | Fatty acid metabolism |
| 10 | 489.2235 | 9.71 | Citicoline | HMDB01413 | 0.66 | 0.003 | 2.60 | 0.001 | 1.46 | Lipid | Glycerophospholipid metabolism |
| 11 | 520.5127 | 9.71 | Lysophosphatidylcholine(18:2) | HMDB10386 | 0.65 | 0.003 | 2.28 | 0.001 | 1.05 | Lipid | Glycerophospholipid metabolism |
| 12 | 566.2283 | 10.37 | Lysophosphatidylethanolamine(24:0) | HMDB11497 | 0.65 | 0.004 | 2.41 | 0.010 | 2.18 | Lipid | Glycerophospholipid metabolism |
| 13 | 241.0052 | 1.98 | Homocarnosine | HMDB00745 | 0.65 | 0.004 | 1.99 | 0.004 | 1.50 | Amino acid | Arginine and proline metabolism |
| 14 | 482.1973 | 10.07 | N-Acetyl-leukotriene E4 | HMDB05084 | 0.63 | 0.005 | 1.37 | 0.015 | 1.10 | Lipid | Fatty acyl metabolism |
| 15 | 430.1670 | 9.21 | Hexadecanedioic acid mono-L-carnitine ester | HMDB00712 | 0.63 | 0.005 | 2.17 | 0.005 | 1.35 | Lipid | Fatty acyl metabolism |
| 16 | 250.0135 | 4.56 | Norepinephrine sulfate | HMDB02062 | 0.63 | 0.006 | 1.65 | 0.005 | 1.48 | Amino acid | Phenylalanine, tyrosine and tryptophan metabolism |
| 17 | 247.1442 | 6.05 | 5,6-Dihydrouridine | HMDB00497 | 0.62 | 0.006 | 2.29 | 0.010 | 1.72 | Nucleotide | Pyrimidine metabolism |
| 18 | 321.0856 | 4.57 | Octanoylglucuronide | HMDB10347 | 0.62 | 0.006 | 1.91 | 0.005 | 2.10 | Carbohydrate | Starch and sucrose metabolism |
| 19 | 295.2055 | 12.78 | Glutamylphenylalanine | HMDB00594 | 0.62 | 0.006 | 1.17 | 0.015 | 1.01 | Amino acid | Glutamate metabolism |
| 20 | 449.2357 | 8.86 | 17-beta-estradiol-3-glucuronide | HMDB06224 | 0.61 | 0.007 | 1.82 | 0.019 | 1.00 | Lipid | Steroid hormone biosynthesis |
| 21 | 635.2993 | 10.40 | Diglyceride(18:4/20:5) | HMDB07346 | 0.61 | 0.007 | 2.11 | 0.002 | 0.93 | Lipid | Glycerolipid metabolism |
| 22 | 396.3471 | 17.96 | 9,12-Hexadecadienoylcarnitine | HMDB13334 | 0.61 | 0.007 | 1.16 | 0.010 | 0.75 | Lipid | Fatty acyl metabolism |
| 23 | 208.0393 | 5.67 | N-Acetyl-L-phenylalanine | HMDB00512 | 0.61 | 0.008 | 1.46 | 0.019 | 0.87 | Amino acid | Phenylalanine metabolism |
| 24 | 243.0839 | 1.90 | Thymidine | HMDB00273 | 0.60 | 0.008 | 2.36 | 0.012 | 2.12 | Nucleotide | Pyrimidine metabolism |
| 25 | 444.2805 | 8.86 | 12-Hydroxy-12-octadecanoylcarnitine | HMDB13154 | 0.60 | 0.008 | 2.01 | 0.003 | 1.83 | Lipid | Fatty acyl metabolism |
| 26 | 244.0871 | 2.51 | Biotin amide | HMDB01458 | 0.59 | 0.009 | 3.05 | 0.004 | 3.63 | Cofactors and vitamins | Biotin metabolism |
| 27 | 440.1771 | 9.40 | Leukotriene E4 | HMDB02200 | 0.59 | 0.010 | 2.14 | 0.003 | 0.89 | Lipid | Arachidonic acid metabolism |
| 28 | 207.0176 | 2.75 | Lipoic acid | HMDB01451 | 0.59 | 0.010 | 1.55 | 0.010 | 1.36 | Cofactors and vitamins | Lipoic acid metabolism |
| 29 | 245.1495 | 8.54 | N-(3-(1-Indol-3-yl)-1-oxo-2-propenyl)glycine | HMDB06005 | 0.59 | 0.010 | 1.69 | 0.015 | 1.08 | Amino acid | Tryptophan metabolism |
| 30 | 269.1261 | 6.03 | Arabinosylhypoxanthine | HMDB03040 | 0.58 | 0.012 | 2.03 | 0.006 | 1.44 | Nucleotide | Purine metabolism |
| 31 | 244.0873 | 1.91 | Cytidine | HMDB00089 | 0.58 | 0.012 | 2.62 | 0.015 | 2.02 | Nucleotide | Pyrimidine metabolism |
| 32 | 301.1797 | 12.65 | Retinoic acid | HMDB01852 | 0.57 | 0.013 | 1.63 | 0.004 | 0.92 | Cofactors and vitamins | Retinol metabolism |
| 33 | 405.2096 | 8.34 | 7a,12a-Dihydroxy-3-oxo-4-cholenoic acid | HMDB00447 | 0.57 | 0.013 | 2.30 | 0.005 | 1.67 | Lipid | Bile acid metabolism |
| 34 | 329.2109 | 12.83 | Monoglyceride(16:1) | HMDB11534 | 0.56 | 0.015 | 1.94 | 0.004 | 1.77 | Lipid | Glycerolipid metabolism |
| 35 | 308.1037 | 1.28 | dCMP | HMDB01202 | 0.56 | 0.016 | 2.86 | 0.005 | 2.76 | Nucleotide | Pyrimidine metabolism |
| 36 | 232.0029 | 5.68 | Isovalerylglutamic acid | HMDB00726 | 0.56 | 0.016 | 1.09 | 0.006 | 0.94 | Amino acid | Glutamate metabolism |
| 37 | 262.0135 | 3.02 | O-Phosphotyrosine | HMDB06049 | 0.56 | 0.017 | 2.04 | 0.012 | 2.02 | Amino acid | Tyrosine metabolism |
| 38 | 349.1985 | 16.08 | Riboflavin reduced | HMDB01557 | 0.55 | 0.017 | 1.23 | 0.008 | 0.48 | Cofactors and vitamins | Riboflavin metabolism |
| 39 | 428.1614 | 9.21 | Stearoylcarnitine | HMDB00848 | 0.55 | 0.017 | 1.91 | 0.001 | 0.86 | Lipid | Fatty acyl metabolism |
| 40 | 303.1954 | 13.35 | Enterodiol | HMDB05056 | 0.55 | 0.018 | 1.76 | 0.006 | 1.25 | Lipid | Steroid metabolism |
| 41 | 336.0151 | 0.90 | Dihydroneopterin phosphate | HMDB06824 | 0.54 | 0.020 | 1.68 | 0.029 | 2.47 | Cofactors and vitamins | Folate metabolism |
| 42 | 413.0438 | 9.88 | dIDP | HMDB03536 | 0.54 | 0.020 | 1.04 | 0.010 | 0.45 | Nucleotide | Purine metabolism |
| 43 | 253.1071 | 11.87 | Deoxyinosine | HMDB00071 | 0.53 | 0.022 | 1.33 | 0.015 | 0.79 | Nucleotide | Purine metabolism |
| 44 | 356.2278 | 7.77 | S-Adenosylmethioninamine | HMDB00988 | 0.53 | 0.023 | 2.34 | 0.002 | 2.27 | Amino acid | Cysteine and methionine metabolism |
| 45 | 338.0679 | 1.46 | Kyotorphin | HMDB05768 | 0.53 | 0.024 | 1.51 | 0.019 | 0.81 | Amino acid | Arginine metabolism |
| 46 | 724.5248 | 20.80 | Phosphatidylethanolamine(18:3/P-18:1) | HMDB09150 | 0.53 | 0.024 | 1.28 | 0.015 | 0.71 | Lipid | Glycerophospholipid metabolism |
| 47 | 868.5838 | 19.11 | Phosphatidylcholine(18:3/24:0) | HMDB08190 | 0.53 | 0.024 | 1.21 | 0.035 | -0.78 | Lipid | Glycerophospholipid metabolism |
| 48 | 386.2205 | 12.18 | 3-Hydroxy-cis-5-tetradecenoylcarnitine | HMDB13330 | 0.53 | 0.024 | 1.39 | 0.029 | 0.38 | Lipid | Fatty acyl metabolism |
| 49 | 761.6537 | 21.44 | Sphingomyelin(d18:0/20:0) | HMDB12090 | 0.53 | 0.024 | 1.14 | 0.002 | 0.73 | Lipid | Sphingolipid metabolism |
| 50 | 756.5906 | 21.75 | Glucosylceramide(d18:1/20:0) | HMDB04973 | 0.53 | 0.024 | 1.66 | 0.023 | -1.51 | Lipid | Sphingolipid metabolism |
| 51 | 169.0356 | 1.47 | Uric acid | HMDB00289 | 0.53 | 0.025 | 1.09 | 0.029 | 0.39 | Nucleotide | Purine metabolism |
| 52 | 261.0265 | 2.49 | 5-Methylthioribose 1-phosphate | HMDB00963 | 0.53 | 0.025 | 2.18 | 0.029 | 1.42 | Amino acid | Cysteine and methionine metabolism |
| 53 | 261.1446 | 6.73 | Glucose 1-phosphate | HMDB01586 | 0.52 | 0.027 | 1.33 | 0.004 | 1.10 | Carbohydrate | Starch and sucrose metabolism |
| 54 | 172.0063 | 6.64 | Tetrahydrodipicolinate | HMDB12289 | 0.52 | 0.029 | 1.47 | 0.043 | 1.10 | Amino acid | Lysine metabolism |
| 55 | 347.2217 | 13.23 | Cortexolone | HMDB00015 | 0.51 | 0.029 | 2.03 | 0.002 | 1.34 | Lipid | Steroid hormone biosynthesis |
| 56 | 380.2130 | 5.60 | S-Lactoylglutathione | HMDB01066 | 0.50 | 0.033 | 1.63 | 0.043 | 1.41 | Carbohydrate | Pyruvate metabolism |
| 57 | 851.6719 | 21.82 | Triglyceride(16:1/16:1/20:4) | HMDB05436 | 0.49 | 0.037 | 1.83 | 0.005 | -0.57 | Lipid | Glycerolipid metabolism |
| 58 | 249.9771 | 3.02 | Pyridoxine 5'-phosphate | HMDB01319 | 0.49 | 0.037 | 1.22 | 0.015 | 1.39 | Cofactors and vitamins | Vitamin B6 metabolism |
| 59 | 361.1832 | 7.77 | Aldosterone | HMDB00037 | 0.49 | 0.039 | 2.12 | 0.008 | 2.09 | Lipid | Steroid hormone biosynthesis |
| 60 | 431.2180 | 9.73 | Monoglyceride(24:6) | HMDB11560 | 0.49 | 0.039 | 1.99 | 0.005 | 0.82 | Lipid | Glycerolipid metabolism |
| 61 | 258.9796 | 2.49 | Imidazoleacetic acid riboside | HMDB02331 | 0.49 | 0.041 | 2.20 | 0.000 | 1.29 | Amino acid | Histidine metabolism |
| 62 | 343.2842 | 16.91 | Monoglyceride(P-18:0e) | HMDB11153 | 0.49 | 0.041 | 1.44 | 0.035 | 1.76 | Lipid | Glycerolipid metabolism |
| 63 | 327.1475 | 16.19 | 6,7-Dimethyl-8-(1-D-ribityl)lumazine | HMDB03826 | 0.47 | 0.048 | 1.30 | 0.023 | 1.06 | Cofactors and vitamins | Riboflavin metabolism |
| 64 | 754.5358 | 19.85 | Phosphatidylcholine(14:0/20:4) | HMDB07883 | -0.47 | 0.049 | 1.73 | 0.035 | -2.82 | Lipid | Glycerophospholipid metabolism |
| 65 | 168.0147 | 1.99 | 2,8-Dihydroxyadenine | HMDB00401 | -0.51 | 0.032 | 1.48 | 0.035 | -0.22 | Nucleotide | Purine metabolism |
| 66 | 834.6367 | 18.97 | Phosphatidylcholine(18:1/22:5) | HMDB08088 | -0.52 | 0.028 | 2.08 | 0.029 | -1.71 | Lipid | Glycerophospholipid metabolism |
| 67 | 506.3844 | 18.47 | Lysophosphatidylethanolamine(20:2) | HMDB11483 | -0.54 | 0.020 | 1.44 | 0.003 | -0.86 | Lipid | Glycerophospholipid metabolism |
| 68 | 205.0287 | 1.51 | L-Tryptophan | HMDB00929 | -0.59 | 0.010 | 1.12 | 0.005 | -0.68 | Amino acid | Tryptophan metabolism |
| 69 | 233.9821 | 1.51 | Dopamine 4-sulfate | HMDB04148 | -0.59 | 0.009 | 1.15 | 0.005 | -0.97 | Amino acid | Tyrosine metabolism |
| 70 | 188.9684 | 1.52 | L-glycyl-L-hydroxyproline | HMDB11173 | -0.61 | 0.008 | 1.43 | 0.010 | -1.41 | Amino acid | Proline metabolism |

Metabolites were identified based on accurate mass data, retention time, experimental MS/MS spectra, and library MS/MS spectra (HMDB, MyCompoundID). A total of 240 metabolites were selected among the meaningful metabolites (*n* = 588, VIP > 1.0, *p* < 0.005) after excluding drugs (*n* = 20), xenobiotics (*n* = 82), differential metabolites at week 0 (*n* = 147), and metabolite identification fit score under 0.9 (*n* = 99). Pearson’s *r* correlation analysis was performed and *p* < 0.05 were listed in the order of correlation coefficient magnitude. Fold change was calculated by dividing the value of metabolite intensity in week 2 of the LCKD by that in week 2 of the GD. *p* < 0.05 represents statistical significance between GD and LCKD at week 2 determined by Mann-Whitney U test. VIP, Variable Importance in Projection; FC, fold change
